# Comparative cardioprotective effects of Kuanxiong Aerosol and its individual components in a rat model of acute myocardial infarction

**DOI:** 10.1080/13880209.2026.2626099

**Published:** 2026-02-15

**Authors:** Kai Qian, Xiao-jing Fan, Xiao-kang Peng, Ye-feng Chen, Meng-meng Xu, Jin-ting Zhang, Yang-yang Zhu, Tao Yang, Yuan-hang Zheng, Yu Tang, Yi Luo

**Affiliations:** ^a^ Postdoctoral Research Center, Zhejiang Sukean Pharmaceutical Co., Ltd, Hangzhou, China; ^b^ Pharmaceutical Informatics Institute, College of Pharmaceutical Sciences, Zhejiang University, Hangzhou, China

**Keywords:** Acute myocardial infarction, volatile oils, sandalwood, galangal, pepper, borneol, cardioprotection

## Abstract

**Context:**

Kuanxiong Aerosol (KXA), composed of volatile oils from sandalwood (*Santalum album L.*), galangal (*Alpiniae Officinarum Rhizoma*), asarum (*Asari Radix*), pepper (*Piper longum L.*), and borneol, is used to treat cardiovascular conditions. However, its scientific basis and the contribution of its individual components remain poorly understood.

**Objective:**

We investigated the chemical composition, dose-dependent efficacy, and cardioprotective effects of KXA and its individual components in a rat acute myocardial infarction (AMI) model.

**Materials and methods:**

KXA and its components were characterized using GC-MS. AMI was induced in rats by left anterior descending (LAD) ligation. Animals were pretreated with low-dose (120 µL/kg) or high-dose (200 µL/kg) KXA, its individual components (120 µL/kg), or isosorbide mononitrate (ISMN; 5 mg/kg). Cardioprotective effects were assessed *via* electrocardiography, TTC staining, histopathology, hemodynamics, and serum cardiac biomarkers.

**Results:**

Low-dose KXA provided significant cardioprotection, improving ST-segment elevation, infarct size, histopathology, hemodynamic, and biochemical markers, with efficacy comparable or more pronounced to ISMN. In contrast, high-dose KXA was less effective. Sandalwood and asarum oils were the primary anti-ischemic agents, while galangal oil, pepper oil, and borneol provided complementary anti-fibrotic and hemodynamic support. However, individual components or high doses exhibited limited efficacy and potential adverse effects.

**Discussion and conclusions:**

KXA’s cardioprotection stems from the synergistic action of its components targeting ischemia, fibrosis, inflammation, and cardiac dysfunction. This study provides evidence supporting the cardioprotective potential of low-dose, multi-component KXA, indicating that such balanced formulations may offer broader benefits than high-dose or single-agent approaches. Further research is needed to elucidate the molecular mechanisms and refine the formulation for clinical translation.

## Introduction

Coronary heart disease, often manifesting as angina pectoris, is a clinical syndrome caused by transient acute myocardial ischemia (AMI) and hypoxia. This condition, characterized by reduced blood flow to the heart and resulting in inadequate oxygen supply to the cardiac tissue, represents one of the most prevalent cardiovascular diseases globally (Mensah et al. 2023). According to the World Heart report 2023, more than half a billion people worldwide continue to be affected by cardiovascular diseases, which accounted for 20.5 million deaths in 2021 (Di Cesare et al. 2024). Ischemic heart disease alone accounts for approximately 42% of all cardiovascular diseases (Woo 2009; Ardjmand et al. 2019). The pathophysiology of myocardial ischemia involves complex mechanisms, including disruptions in energy metabolism, excessive generation of reactive oxygen species, calcium overload, inflammatory cell ­infiltration, and ultimately, cardiomyocyte apoptosis and necrosis (Ahmad et al. 2010; Zhao et al. 2023). If not treated promptly, these pathological processes can culminate in irreversible myocardial damage, underscoring the critical need for effective interventions.

Consequently, the prevention and treatment of MI has become a major focus of clinical and research attention. Current treatment options for angina include pharmacological therapies such as β-blockers, calcium channel blockers, nitrates, and endogenous nitric oxide donors (Chong et al. 2016; Santucci et al. 2020), as well as surgical interventions like revascularization and spinal cord stimulation (Honda 2021). However, despite the wide range of available treatments, approximately 5%–10% of patients with refractory angina continue to experience persistent symptoms, and cardiovascular disease with angina remains a significant and unresolved public health concern, driving the search for novel therapeutic strategies (Makowski et al. 2020).

Given the multifaceted nature of myocardial ischemia, it is increasingly recognized that multi-mechanism therapeutic approaches are necessary. Traditional Chinese medicine (TCM), renowned for its holistic approach and multi-target, multi-pathway mode of action, offers distinct advantages for the treatment of myocardial ischemia. KuanXiong Aerosol (KXA), a Chinese herbal medicine formula which was optimized by Academician Chen Keji and Professor Guo Shikui, follows the traditional principle of ‘Fangxiang Wentong,’ which involves dredging blood vessels and resuscitating life with pungent and warm-natured drugs (Lu et al. 2021). Clinical studies have shown that KXA has similar efficacy to nitroglycerin tablets in rapidly alleviating coronary angina, but with fewer adverse reactions. These advantageous features of KXA, including its rapid onset, ease of use, and low toxicity, have contributed to its widespread integration into clinical practice (Li et al. 2014; Zhuang et al. 2020; Huang et al. 2024).

KXA comprises a blend of four volatile oils derived from sandalwood (*Santalum album L.*), galangal (*Alpiniae Officinarum Rhizoma*), asarum (*Asari Radix*), and pepper (*Piper longum L.*), combined with borneol (Zhang et al. 2021). This formulation has demonstrated efficacy against myocardial ischemia, inflammation, and oxidative stress in various studies (Diwan et al. 2013; Basri et al. 2017, Bommareddy et al. 2019; Liu et al. 2021). Recent experimental investigations have revealed that KXA exerts its cardioprotective effects through multiple mechanisms, including the modulation of the TRPV1 channel (Chen et al. 2025a), inhibition of the mitogen-activated protein kinase (MAPK) pathway (Lu et al. 2021), regulation of calcium homeostasis (Lu et al. 2022), and anti-inflammatory, antioxidant, and anti-apoptotic activities (Wu et al. 2020; Chen et al. 2025b).

Although the overall therapeutic effects of KXA have been well documented, the relative contribution of each herbal component to its holistic pharmacological activity remains largely unclear. The TCM theory emphasizes the synergistic effects achieved through herbal combinations, suggesting that the whole formulation is intended to offer enhanced therapeutic benefits compared to individual components used separately. However, a critical knowledge gap exists, as systematic evaluations comparing the efficacy of the complete KXA formulation and that of its individual herbal components in myocardial ischemia models are lacking.

Therefore, this study was designed to address this gap by comparing the pharmacological effects of KXA and its individual herbal components in a rat model of myocardial ischemia. Various parameters, including electrocardiographic changes, myocardial enzyme leakage, histopathological alterations, hemodynamics, and serum biomarkers, were assessed. By elucidating the differential effects of the complete formulation versus the individual components, this study aims to provide valuable insights into the scientific rationale behind the traditional formula composition and will potentially guide the future optimization of KXA for enhanced therapeutic outcomes in myocardial ischemia.

## Materials and methods

### Reagents

Isosorbide mononitrate tablets (ISMN) (Lot: H10940039) were purchased from Lunan Better Pharmaceutical Co., Ltd. (Shandong, China). KXA (lot: 22408006) was provided by Zhejiang Sukean Pharmaceutical Co., Ltd. Myocardial enzyme assay kits for CK-MB (105-000459-00), CK (105-000458-00), LDH (105-000446-00), and AST (105-000443-00) were purchased from Shenzhen Mindray Bio-Medical Electronics Co. (Shenzhen, China). Triphenyl tetrazolium chloride (TTC, Cat: G3005) and a Modified Masson’s trichrome staining kit (Cat: G1346) were purchased from Beijing Solarbio Science & Technology Co., Ltd. Absolute ethanol (Cat: 100092683) was purchased from Sinopharm Chemical Reagent Co., Ltd. (Shanghai, China). The hematoxylin and eosin (H&E) kit (Cat: C0105M) and acid alcohol slow differentiation solution (Cat: C0161M) were purchased from Beyotime Biotech, Inc.

### Preparation and formulation of KXA components

**Figure 1.**
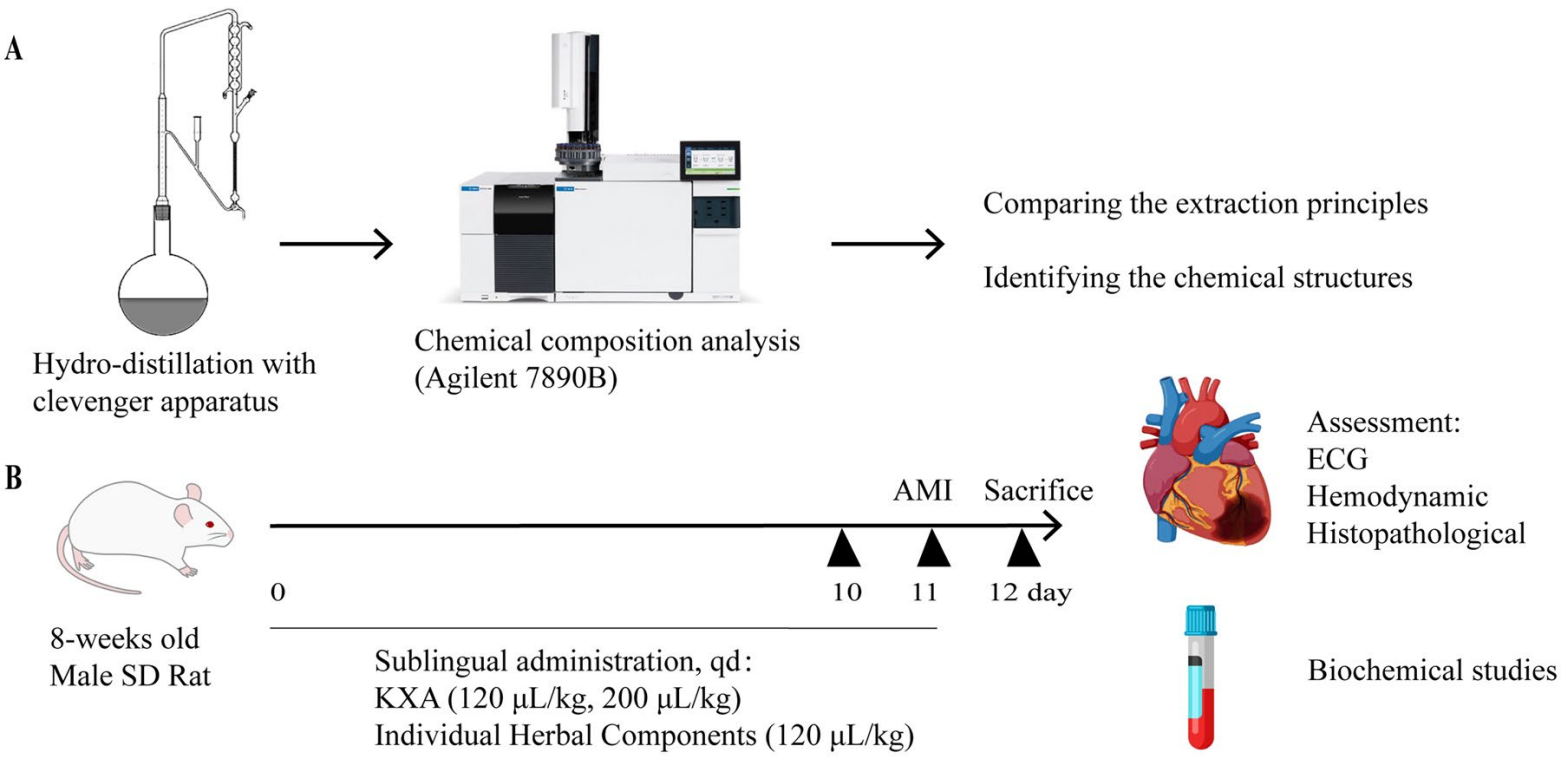
Schematic of the experimental protocol.

Volatile oils were extracted from the four herbal components *via* Hydro-distillation with clevenger apparatus steam distillation. Raw herbal materials were first crushed or sliced, as appropriate. The processed herb was then placed in a distillation tank with 0.5–2 times its weight in water. The mixture was heated to boiling, and steam distillation was conducted for a duration specific to each herb: at least 50 h for sandalwood, 7 h for pepper and galangal, and 5 h for asarum. The collected volatile oil was used for subsequent formulation (Figure 1A).

The extracted volatile oils and solid borneol were then formulated into ethanolic solutions corresponding to the established component ratios of KXA in the Chinese Pharmacopeia. Sandalwood oil was mixed with ethanol at a ratio of 1:7.93 (0.7 mL oil to 5.55 mL ethanol), to yield an 11.2% (v/v) solution. Pepper oil was combined with ethanol at a 1:40.67 ratio (0.15 mL oil to 6.1 mL ethanol) to prepare a 2.4% (v/v) solution. Galangal oil was blended with ethanol at a 1:18.53 ratio (0.32 mL oil to 5.93 mL ethanol) to obtain a 5.12% (v/v) solution. Asarum oil was mixed with ethanol at a 1:24.08 ratio (0.25 mL oil to 6.02 mL ethanol) to form a 3.68% (v/v) solution. Borneol was combined with ethanol at a 1:27.8 ratio (0.1 g borneol to 2.78 mL ethanol), resulting in a final concentration of 36 mg/mL.

All solutions were prepared by adding a specified amount of oil or borneol to ethanol in clean glass vials, followed by vortexing or gentle shaking until complete dissolution was achieved. Ethanol was used as a solvent to ensure complete dissolution and homogeneity of the mixture. The proportions strictly followed the official formulation of KXA to maintain consistency. All the solutions were freshly prepared before use, stored at room temperature, and protected from light.

### GC-MS quantification

Headspace gas chromatography–mass spectrometry (HS-GC-MS) was used to identify the volatile components of the four extracted volatile oils used in KXA (Figure 1A). The analysis was conducted using an Agilent 7890B gas chromatograph coupled with a 5977B MSD mass spectrometer, equipped with an Agilent HP-5MS capillary column (30 m × 0.25 mm i.d., 0.25 μm film thickness; 5% phenyl methyl siloxane, Agilent 19091S-433). The GC oven temperature was initially set at 40 °C and held for 3 min, then ramped to 300 °C at a rate of 5 °C/min, with a final hold of 3 min. The maximum oven temperature was 325 °C. A 1 µL sample was injected using helium as the carrier gas at a flow rate of 1.0 mL/min, decreasing to 0.5735 mL/min during the run. The front SS inlet was operated in split mode (280 °C, 7.0699 psi, split ratio 40:1), whereas the rear SS inlet was operated in pulsed splitless mode (25 psi, cutoff time 0.5 min). A gas sampling valve (GSV) with a 1 mL loop was used with a sample filling and injection time of 0.5 min. The data were acquired at 50 Hz with a solvent delay of 3 min. The ion source and quadrupole temperatures were set at 230 °C and 150 °C, respectively. These parameters enabled the efficient identification and characterization of the volatile oils in the samples. It should be noted that GC–MS analysis provides qualitative and semi-quantitative information on volatile constituents, however, the relative abundance of individual components may vary depending on raw material sources and extraction conditions, which is an inherent characteristic of herbal volatile oils.

### Animal experiments

All animal experiments were performed according to the National Institutes of Health Guide for the Care and Use of Laboratory Animals and the ARRIVE guidelines. This study was approved by the Ethics Committee of Zhejiang Sukean Pharmaceutical Co., Ltd. Medical Laboratory Animal Center (Approval No. ZJSKA-IACUC-240603). All efforts were made to minimize animal suffering.

A total of 120 male Sprague-Dawley rats, specific pathogen-free (SPF), weighing 220–250 g, were provided by Zhejiang Vital River Experimental Animal Technology Co., Ltd. (Certification No. SCXK-(Zhe) 2024-0001, Quality Qualification Certificate No. 20240918Aazz0619999517). Animals were raised in the SPF houses of the Zhejiang Sukean Pharmaceutical Co., Ltd. Medical Laboratory Animal Center. The temperature of SPF houses was maintained at 22–24 °C, with a relative humidity of 40–70%, and a 12 h light–dark cycle. Animals were administered sterile water and rodent food *ad libitum*. All experiments began after a 1-week acclimatization period. All animal procedures and data collection were performed between June 2024 and June 2025.

### Experimental design and drug administration

After acclimatization, the rats were randomly allocated into 10 groups (*n* = 12 each): (1) Control (sham operation), (2) Model (AMI + vehicle), (3) ISMN (positive control), (4) low-dose (120 µL/kg) KXA (KXA-L), (5) high-dose (200 µL/kg) KXA (KXA-H), (6) Sandalwood oil, (7) Pepper oil, (8) Asarum oil, (9) Galangal oil, and (10) Borneol.

The KXA doses were selected based on clinical dose equivalence. The low dose (120 µL/kg) corresponded to approximately three times, and the high dose (200 µL/kg) to approximately five times, the clinically used human dose after interspecies dose conversion, allowing evaluation of both clinically relevant exposure and dose-dependent effects.

For 10 consecutive days, treatments were administered as follows: ISMN was administered orally at 5 mg/kg/day. The KXA-L and KXA-H groups, equivalent to three and five times the daily human dose, respectively, received their treatments by sublingually. The individual component groups (Sandalwood, Pepper, Asarum, Galangal, Borneol) were administered their respective prepared solutions by gavage at doses equivalent to their concentration in the KXA-H formulation. The Control and Model groups were treated with equal volumes of distilled water by gavage.

### Induction of AMI

On day 11, one hour after the final administration, AMI was induced surgically as previously described (Lindsey et al. 2018). Rats were anesthetized with 2–3% isoflurane to ensure adequate sedation and analgesia. A left thoracotomy was performed in the fourth intercostal space, and the left anterior descending (LAD) coronary artery was ligated with a 6-0 silk suture approximately 2 cm from its origin. Successful AMI induction was confirmed by the immediate appearance of pallor in the anterior left ventricular wall and a significant ST-segment elevation in Lead II ECG. Rats in the sham group underwent the same surgical procedure without LAD ligation. Post-operative care included regular monitoring.

### Electrocardiogram (ECG) and hemodynamic assessment

ECG recordings were obtained using a BL-420N system (Chengdu Taimeng Technology Co., Ltd., China) with needle electrodes placed on the right foreleg and both hind legs. Lead II ECGs were recorded at baseline (before coronary artery ligation) and at 5, 15, 30, 45, 60, 90, 120, and 180 min, and finally at 24 h after ligation. ST-segment amplitude and heart rate (HR) were measured at each time point.

At the 24 h endpoint, prior to euthanasia, hemodynamic parameters were measured. After anesthesia, a PE50 catheter filled with 1% sodium heparin was inserted into the left ventricle *via* the right common carotid artery. Once the signal stabilized, hemodynamic signals, including left ventricular systolic pressure (LVSP), left ventricular end-diastolic pressure (LVEDP), maximum rate of pressure rise (Max dP/dt), and maximum rate of pressure fall (Min dP/dt), were measured for 30 s using a BL-420N Biological Signal Acquisition System.

### Sample collection and infarct area assessment

Following hemodynamic measurement at the 24 h endpoint, the animals were euthanized by inhalation of 5% isoflurane. Terminal blood samples (2–3 mL) were collected from the abdominal aorta for biochemical assays.

The hearts were then rapidly removed and sliced transversely into five sections (1 mm thick), starting from the apex. The slices were incubated with 1% 2,3,5-Triphenyltetrazolium chloride (TTC) staining at 37 °C for 10 min in the dark. Viable myocardial tissue stained red, while the infarct area remained unstained (pale white). The infarct size was expressed as the weight ratio of the infarct area to the whole heart (Infarct Area/Whole Heart × 100%).

### Histopathological evaluation

Cardiac apex tissue samples were fixed in 4% paraformaldehyde for at least 24 h, dehydrated in a graded ethanol series, and embedded in paraffin. Sections (4 μm thick) were cut for staining. H&E staining was performed to assess histopathological changes in myocardial structure. For Masson’s trichrome staining, sections were prepared and stained similarly using a commercial kit according to the manufacturer’s standard procedures to evaluate the degree of cardiac fibrosis. The slides were examined under a light microscope (Nikon DS-Fi2, Japan).

### Biochemical studies

Blood samples, collected from the abdominal aorta after occlusion of the LAD for 24 h at the experimental endpoint, were centrifuged at 3000 ×g for 10 min at 4 °C to obtain serum. Serum levels of the cardiac injury markers creatine kinase (CK), lactate dehydrogenase (LDH), and aspartate aminotransferase (AST) were measured using an automatic biochemical analyzer (Mindray BS-240, Shenzhen, China) following the manufacturer’s instructions.

### Statistical analysis

All data are expressed as mean ± SEM. Statistical comparisons among multiple groups were conducted using one-way analysis of variance (ANOVA), followed by Tukey’s post hoc test for multiple comparisons when appropriate with GraphPad Prism (Version 10.1.2). A *p* value < 0.05 was considered statistically significant.

## Results

### Identification of major components of four volatile oils

**Figure 2.**
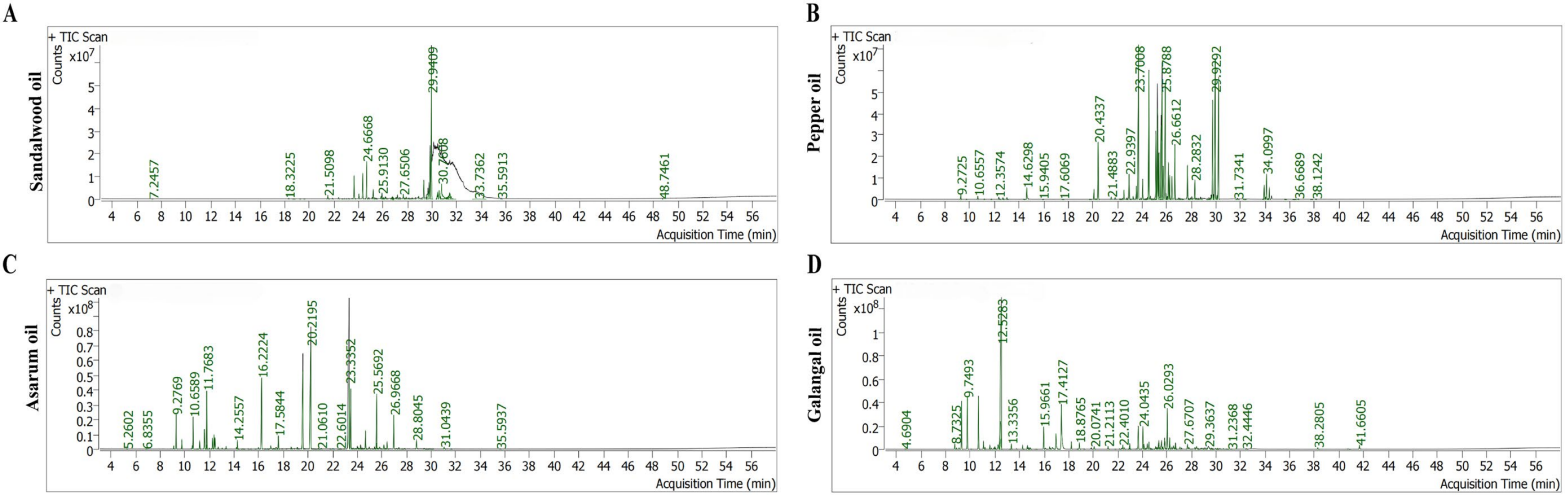
GC-MS analysis chromatogram of the four volatile oils. Representative GC-MS chromatograms of (A) sandalwood, (B) galangal, (C) asarum, and (D) pepper oils.

KXA is an oily substance with a volatile and aromatic odor. GC-MS analysis of sandalwood, galangal, asarum, and pepper oils revealed that each volatile oil exhibited a unique chemical signature, defined by both the type and proportion of its dominant compounds (Figure 2). For clarity and comparability, Tables 1–4 present only the top ten most abundant constituents identified in each volatile oil. The full GC–MS compound lists and relative abundances are provided in the Supplementary Tables S1–S4. Sandalwood oil was distinctively rich in α-santalol (23.94%), with notable contributions from β-santalol (a combined ∼14% considering confirmed isomers) and its ester derivative, (+)-epi-β-santalyl acetate (4.50%); these major sesquiterpene alcohols are key to sandalwood’s unique woody scent and are pivotal for its medicinal and perfumery value (Table 1). Pepper oil, by comparison, is dominated by caryophyllene (9.58%), humulene (7.23%), β-bisabolene (6.36%), (Z)-3-heptadecene (8.30%), and germacrene D (5.59%), highlighting the sesquiterpene-rich pattern and slightly peppery, woody fragrance typical of pepper species (Table 2). Asarum oil features a high proportion of safrole (17.78%), alongside high contents of 2-methoxy-6-methyl-cyclohexa-2,5-dienecarboxamide (11.86%), 2,4-cycloheptadien-1-one, 2,6,6-trimethyl-(8.66%), and benzene, 1,2,3-trimethoxy-5-methyl-(8.49%), representing a typical phenylpropanoid and aromatic ether chemical fingerprint associated with asarum’s pungent, slightly medicinal scent and bioactivity (Table 3). Galangal oil is characterized by a high content of eucalyptol (30.01%), with significant levels of α-terpineol (7.65%), D-limonene (5.66%), camphene (4.98%), and α-pinene (4.69%), reflecting its typical spicy and camphoraceous aroma profile (Table 4). The observed variation in these main components offers a plausible mechanistic link to the distinct pharmacological effects of the oils observed in the subsequent AMI pharmacodynamic experiments.

**Table 1.** Identification of major components of sandalwood oil.

No.	RT	Compound Name	CAS#	Formula	Area MI	Match Score	Area%-T	Area%-M
1	23.6467	Tricyclo[2.2.1.0(2,6)]heptane, 1,7-dimethyl-7-(4-methyl-3-pentenyl)-, (-)	512-61-8	C_15_H_24_	24,314,997	99.4	3.93	16.4
2	24.3445	Bicyclo[2.2.1]heptane, 2-methyl-3-methylene-2-(4-methyl-3-pentenyl)-, (1S-endo)	25532-78-9	C_15_H_24_	27,925,138	99.3	4.51	18.83
3	24.6668	Bicyclo[2.2.1]heptane, 2-methyl-3-methylene-2-(4-methyl-3-pentenyl)-, (1S-endo)	25532-78-9	C_15_H_24_	41,088,322	98.4	6.63	27.71
4	29.3088	Cyclobuta[1,2:3,4]dicyclooctene, 1,2,5,6,6a,6b,7,8,11,12,12a,12b-dodecahydro-, (6a.alpha.,6b.alpha.,12a.beta.,12b.beta.)	61233-68-9	C_16_H_24_	21,345,515	84.8	3.45	14.39
5	29.831	(E)-5-((1R,3R,6S)-2,3Dimethyltricyclo[2.2.1.02,6]heptan-3-yl)-2methylpent-2-enal	19903-70-9	C_15_H_22_O	52,241,706	93.2	8.43	35.23
6	29.9409	.alpha.-Santalol	115-71-9	C_15_H_24_O	148,304,113	99.2	23.94	100
7	30.5594	(+)-Epi-.beta.-santalyl acetate	41414-75-9	C_17_H_26_O_2_	27,898,968	83.2	4.5	18.81
8	30.7608	beta.-Santalol	77-42-9	C_15_H_24_O	44,416,160	85.9	7.17	29.95
9	31.3008	1,2-Benzenediol, O-(4-methylbenzoyl)-O’propoxycarbonyl	1010329-75-8	C_18_H_18_O_5_	27,444,429	73.6	4.43	18.51
10	31.4174	beta.-Santalol	77-42-9	C_15_H_24_O	29,200,415	77.9	4.71	19.69

**Table 2.** Identification of major components of pepper oil.

No.	RT	Compound Name	CAS#	Formula	Area MI	Match Score	Area%-T	Area%-M
1	23.7008	Caryophyllene	87-44-5	C_15_H_24_	229,466,888	99.8	9.58	100
2	24.5479	Humulene	6753-98-6	C_15_H_24_	173,232,689	97.7	7.23	75.49
3	24.5488	(1S,2E,6E,10R)-3,7,11,11Tetramethylbicyclo[8.1.0]undeca-2,6-diene	24703-35-3	C_15_H_24_	121,003,934	80.6	5.05	52.73
4	25.2374	Germacrene D	23986-74-5	C_15_H_24_	133,893,272	93.1	5.59	58.35
5	25.5388	1,3-Cyclohexadiene, 5-(1,5-dimethyl-4-hexenyl)-2methyl-, [S-(R*,S*)]	495-60-3	C_15_H_24_	97,495,596	92	4.07	42.49
6	25.6235	Pentadecane	629-62-9	C_15_H_32_	165,919,453	82.3	6.92	72.31
7	25.8788	β-Bisabolene	495-61-4	C_15_H_24_	152,274,201	98.9	6.36	66.36
8	29.7299	3-Heptadecene, (Z)	1000141-67-3	C_17_H_34_	130,026,752	97.9	5.43	56.66
9	29.9292	3-Heptadecene, (Z)	1000141-67-3	C_17_H_34_	198,841,248	97.8	8.3	86.65
10	30.2087	Heptadecane	629-78-7	C_17_H_36_	172,394,124	99.5	7.2	75.13

**Table 3.** Identification of major components of asarum oil.

No.	RT	Compound Name	CAS#	Formula	Area MI	Match Score	Area%-T	Area%-M
1	9.2769	alpha.-Pinene	80-56-8	C_10_H_16_	53,606,563	99.1	3.36	18.92
2	10.6589	Bicyclo[3.1.1]heptane, 6,6-dimethyl-2-methylene-, (1S)	18172-67-3	C_10_H_16_	51,136,901	98.8	3.21	18.05
3	11.7683	3-Carene	13466-78-9	C_10_H_16_	97,489,367	98.5	6.12	34.4
4	16.2224	2,4-Cycloheptadien-1-one, 2,6,6-trimethyl	503-93-5	C_10_H_14_O	138,118,344	99.3	8.66	48.74
5	19.5738	2-Methoxy-6-methyl-cyclohexa-2,5-dienecarboxamide	1000189-91-0	C_9_H_13_NO_2_	189,053,975	81.7	11.86	66.72
6	20.2195	Safrole	94-59-7	C_10_H_10_O_2_	283,366,101	77.5	17.78	100
7	23.3352	Benzene, 1,2,3-trimethoxy-5-methyl	6443-69-2	C_10_H_14_O_3_	135,280,351	93	8.49	47.74
8	23.4648	Benzene, 1,2,3-trimethoxy-5-methyl	6443-69-2	C_10_H_14_O_3_	109,935,917	95.7	6.9	38.8
9	25.5692	Asaricin	18607-93-7	C_11_H_12_O_3_	101,352,892	95.3	6.36	35.77
10	26.9668	Benzene, 1,2,3-trimethoxy-5-(2-propenyl)	487-11-6	C_12_H_16_O_3_	59,861,179	99	3.76	21.13

**Table 4.** Identification of major components of galangal oil.

No.	RT	Compound Name	CAS#	Formula	Area MI	Match Score	Area%-T	Area%-M
1	9.283	α-Pinene	80-56-8	C_10_H_16_	102,616,685	99	4.69	15.64
2	9.7493	Camphene	79-92-5	C_10_H_16_	108,857,441	99.2	4.98	16.59
3	10.6691	Bicyclo[3.1.1]heptane, 6,6-dimethyl-2-methylene-, (1S)	18172-67-3	C_10_H_16_	119,137,245	98.6	5.45	18.16
4	12.4584	D-Limonene	5989-27-5	C_10_H_16_	123,797,935	83.3	5.66	18.87
5	12.5007	2-Butanone	78-93-3	C_4_H_8_O	57,426,243	82.5	2.63	8.75
6	12.5283	Eucalyptol	470-82-6	C_10_H_18_O	656,186,341	93.9	30.01	100
7	15.9661	(+)-2-Bornanone	464-49-3	C_10_H_16_O	47,630,835	99.8	2.18	7.26
8	17.4127	α-Terpineol	98-55-5	C_10_H_18_O	167,195,625	98.2	7.65	25.48
9	23.6672	Caryophyllene	87-44-5	C_15_H_24_	53,075,594	97.9	2.43	8.09
10	26.0293	Naphthalene,1,2,3,4,4a,5,6,8a-octahydro-7-methyl-4methylene-1-(1-methylethyl)-, (1.alpha.,4a.beta.,8a.alpha.)	39029-41-9	C_15_H_24_	91,850,919	99.2	4.2	14

### Effects on ST-segment changes in an AMI rat model

**Figure 3.**
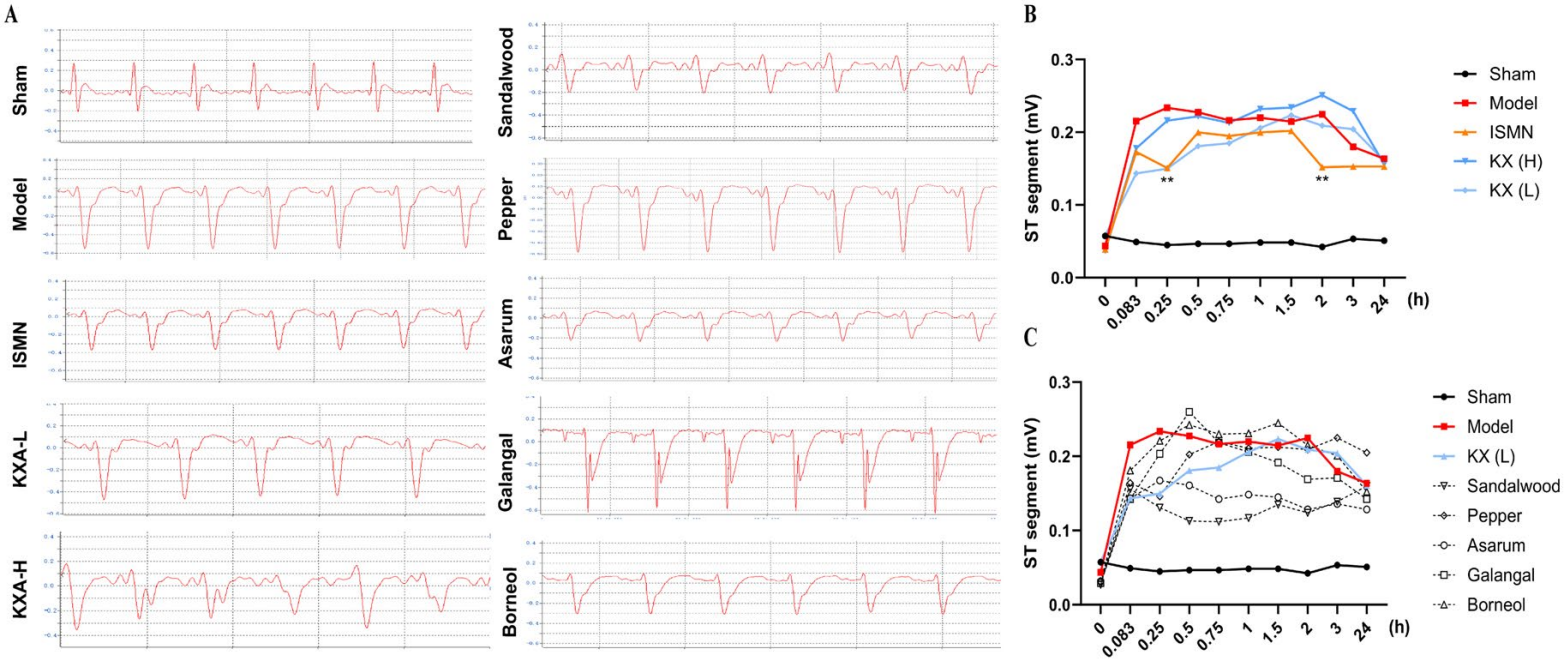
KXA-L attenuates LAD-induced ST-segment elevation predominantly through sandalwood and asarum oils. Effects of KXA and its individual herbal components on electrocardiographic ST-segment elevation in a rat model of acute myocardial infarction induced by left anterior descending (LAD) coronary artery ligation (*n* = 6 per group). (A) Representative ECG traces showing ST-segment changes in each group. (B) Quantitative analysis of ST-segment elevation following treatment with KXA and the positive control isosorbide mononitrate (ISMN). (C) Comparison of ST-segment elevation among groups treated with individual volatile oil components. Data are presented as mean ± SD. *p < 0.05, **p < 0.01 vs. model group.

Electrocardiographic ST-segment analysis was performed to evaluate the effects of KXA and its individual herbal components on myocardial ischemia following LAD ligation in a rat model of AMI (Figure 3A). The KXA-L group demonstrated a rapid and significant improvement in ST-segment elevation at both 5 and 15 min post-ligation, with efficacy comparable to that of the positive control drug, isosorbide mononitrate (ISMN) (*p* < 0.01) (Figure 3B). Notably, at the 5-min time point, the ameliorative effect of KXA-L was greater than that of ISMN. In contrast, the KXA-H group did not show a similar significant improvement.

Among the groups treated with individual herbal components, sandalwood oil and asarum oil produced marked reductions in ST-segment elevation, whereas pepper longum oil, galangal oil, and borneol showed no significant effects on the ST-segment (Figure 3C). These findings suggest that the beneficial effects of KXA on ST-segment changes in this AMI model are primarily attributable to the action of sandalwood and asarum oils.

### Myocardial infarct size assessment by TTC staining

**Figure 4.**
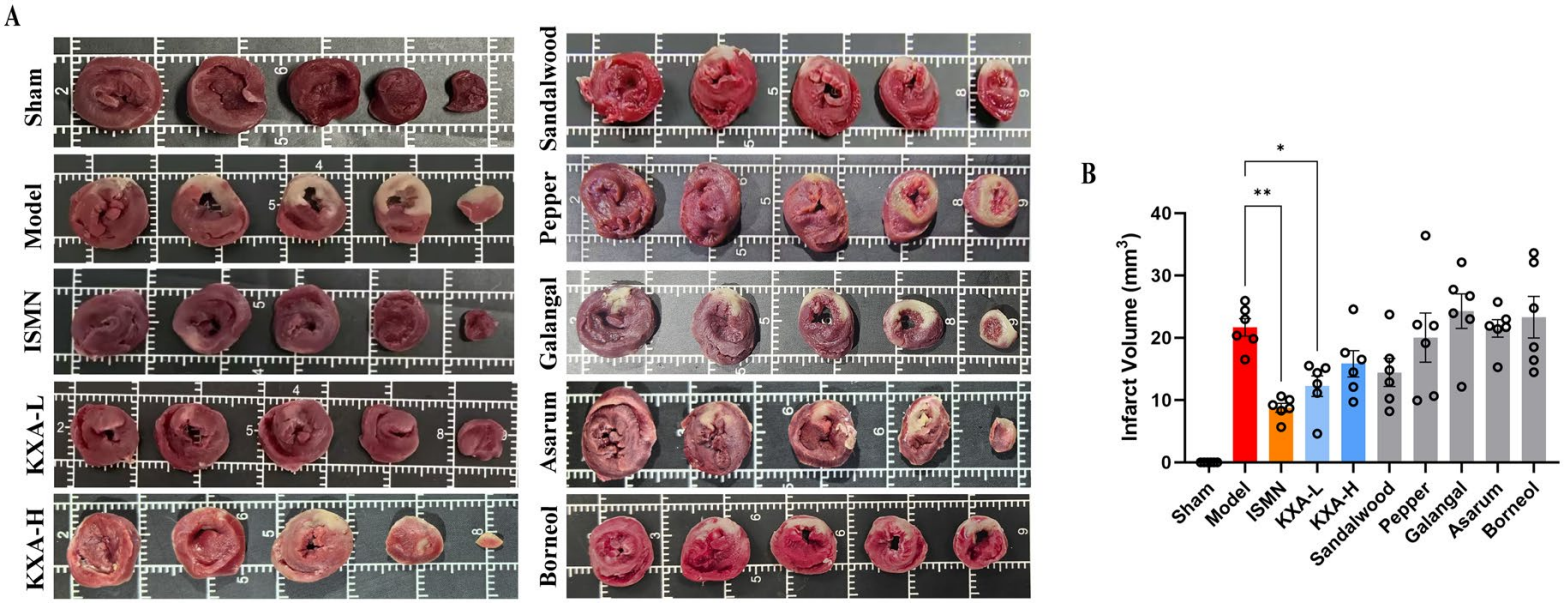
KXA reduces myocardial infarct size predominantly through its sandalwood oil component. Myocardial infarct size in rats subjected to cardiac ischemia injury with KXA and its individual herbal components administered prior to ischemia (*n* = 6 per group). (A) Representative photographs of TTC staining. (B) Global infarct size were quantitatively analyzed. Data are presented as the mean ± SD. *p < 0.05 vs model, **p < 0.01 vs model. TTC, 2,3,5-triphenyltetrazolium chloride.

TTC staining demonstrated that the administration of KXA-L significantly reduced the myocardial ischemic area compared to the model group (*p* < 0.05; Figure 4A,B). Nevertheless, this cardioprotective effect was slightly less pronounced than that of the positive control, ISMN, which achieved a more marked reduction in infarct size (*p* < 0.01). Consistent with the ST-segment analysis, the KXA-H group did not exhibit greater efficacy compared to the low-dose group in reducing infarct size.

Among the groups receiving individual herbal constituents, sandalwood oil produced the most pronounced reduction in the ischemic area, surpassing all other single-component interventions (Figure 4A,B). The remaining four constituents did not elicit statistically significant improvements in myocardial infarct size. Collectively, these findings suggest that sandalwood oil was the principal contributor to the infarct-sparing activity of KXA against myocardial ischemia.

### Comparative pathological evaluation of KXA and its volatile oils

**Figure 5.**
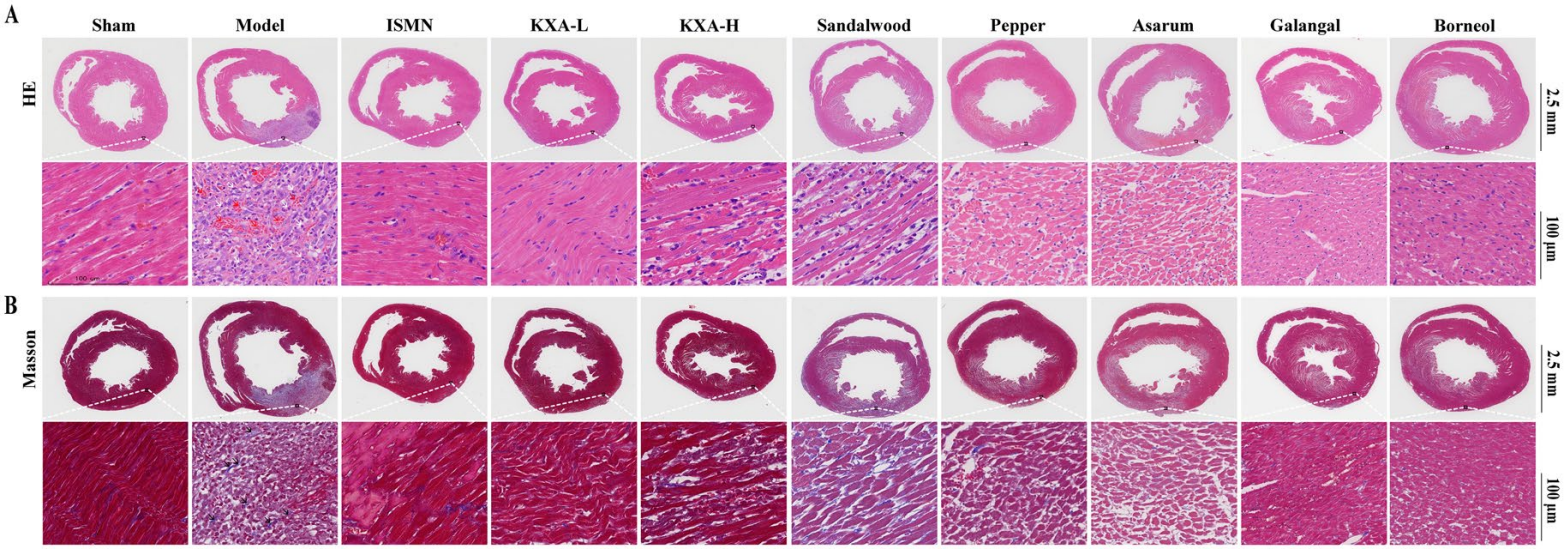
KXA-L alleviated myocardial damage and fibrosis compared with the model group. Histopathological evaluation of KXA and its components in LAD-induced myocardial injury, the morphological and pathological changes of the myocardial tissues in the different groups (*n* = 6 per group) were observed with (A) H&E and (B) Masson at 2.5 mm (10×) and 100 μM (200×) magnifications, respectively.

Histopathological analysis using H&E and Masson’s trichrome staining demonstrated that KXA and its individual herbal components exerted a protective effect against myocardial injury and fibrosis. In the model group, myocardial cells exhibited structural disarray, nuclear pyknosis, karyorrhexis, or disappearance, as well as loss of membrane integrity in the infarcted area (white arrows), accompanied by significant inflammatory cell infiltration (red arrows, Figure 5A). Masson’s trichrome staining revealed marked myocardial fibrosis and collagen deposition (black arrows, Figure 5B). After treatment with KXA, the myocardial cell arrangement was more orderly, the membrane structure was better preserved, the inflammatory response was alleviated, and the degree of myocardial fibrosis and collagen deposition was reduced, indicating an improvement in myocardial injury and fibrosis (Figure 5A,B). A comparison between the different dosage groups revealed that KXA-L was more effective than KXA-H, as evidenced by a more pronounced restoration of myocardial structure and lower levels of inflammation and fibrosis.

Among the groups treated with individual components, the sandalwood oil group exhibited the most extensive myocardial fibrosis, followed by the pepper oil and asarum oil groups. Galangal oil and borneol were relatively more effective in reversing fibrosis; however, the galangal oil group showed residual inflammatory cell infiltration in the myocardial interstitium, whereas the borneol group exhibited unclear or absent myocardial striations, wavy changes, and myocardial cell swelling with increased cytoplasmic eosinophilia (Figure 5).

Overall, these results suggest that KXA and its volatile oils can mitigate myocardial injury and fibrosis; however, the protective effects and potential adverse reactions vary among the different components, with the KXA-L showing a more pronounced efficacy.

### Effects on hemodynamic parameters

**Figure 6.**
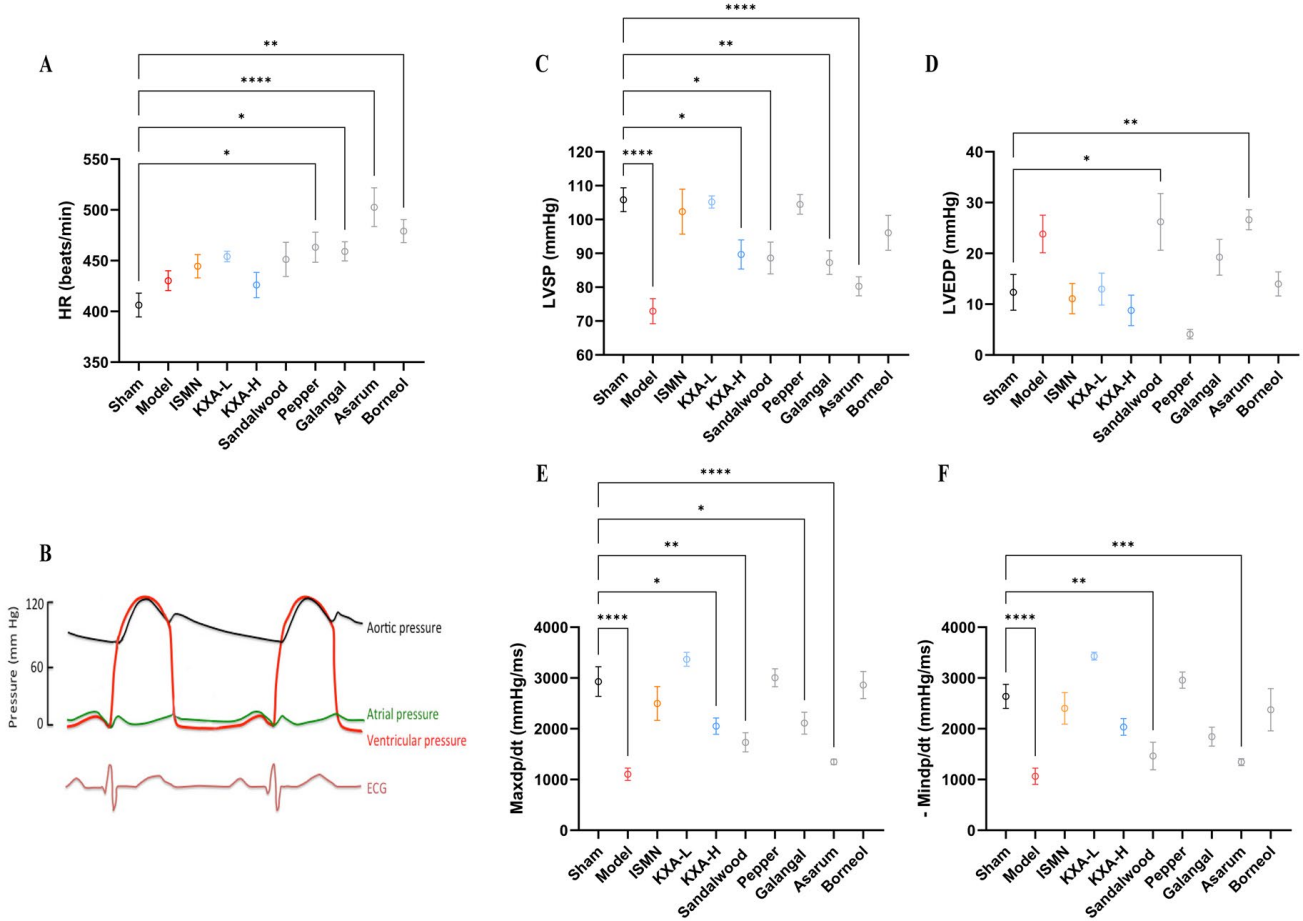
KXA-L improves cardiac hemodynamics in AMI rats without affecting heart rate. Heart rate (HR, A) and hemodynamic parameters, including left ventricular systolic pressure (LVSP, C), left ventricular end-diastolic pressure (LVEDP, D), maximum rate of pressure rise (Max + dP/dt, E), and minimum rate of pressure decline (Min –dP/dt, F), in AMI rats treated with different doses of KXA and its individual herbal components (*n* = 8 per group). The data are presented as the mean ± SD. *p < 0.05 vs. sham, **p < 0.01 vs. sham, ***p < 0.001 vs. sham, ****p < 0.0001 vs. sham.

In this study, we systematically evaluated the effects of isosorbide mononitrate (ISMN), KXA, and their main herbal components on cardiac hemodynamics. The results showed that neither ISMN nor KXA significantly affected the systemic heart rate, with values comparable to those of the model and sham groups. In contrast, the pepper oil, galangal oil, asarum oil, and borneol groups exhibited a marked increase in heart rate, suggesting that these individual components may possess stimulatory or sympathomimetic effects on the cardiovascular system, possibly enhancing cardiac excitability or stimulating adrenergic pathways. (Figure 6A).

As a key indicator of ventricular contractility, left ventricular systolic pressure (LVSP) represents the peak ventricular pressure during systole, which must exceed aortic pressure to open the aortic valve and eject blood (Figure 6B). The difference between the LVSP and aortic pressure reflects the ability of the ventricle to overcome afterload. In this study, LVSP was significantly lower in the model group than in the sham group and was also markedly reduced in the sandalwood oil, galangal oil, and asarum oil groups (Figure 6C). Both ISMN and KXA-L (120 µL/kg) significantly increased the LVSP, indicating improved ventricular systolic function. Unexpectedly, the KXA-H (200 µL/kg) did not outperform the low-dose group and caused a further significant decrease in LVSP (*p* < 0.05), suggesting a potential negative inotropic effect at higher doses.

Left ventricular end-diastolic pressure (LVEDP) represents the pressure in the ventricle at the end of diastole and is influenced by the volume of ventricular filling and compliance. LVEDP is also affected by atrial pressure, with elevated atrial pressure increasing the ventricular filling pressure (Figure 6B). In this study, KXA effectively alleviated ventricular preload, with LVEDP levels similar to those in the sham group, indicating preserved ventricular compliance. Pepper oil and borneol showed similar effects (Figure 6D). Conversely, sandalwood and asarum oils significantly increased LVEDP, indicating elevated atrial pressure and reduced ventricular compliance, which may be detrimental to the recovery of diastolic function (Figure 6D).

Regarding myocardial contractility and diastolic function, as assessed by maximum rate of pressure rise (Max dP/dt) and minimum rate of pressure decline (Min dP/dt), the KXA-H, sandalwood oil, galangal oil, and asarum oil groups failed to restore the isovolumetric contraction rates to normal, with the asarum oil group being particularly affected. In contrast, the pepper oil and borneol groups showed greater improvements in both parameters. Notably, KXA-L demonstrated a numerically greater enhancement of both Max dP/dt and Min dP/dt compared to ISMN, indicating a more pronounced overall improvement in both systolic and diastolic function (Figure 6E,F). In summary, the KXA-L group exhibited the most favorable effects on multiple cardiac function parameters, whereas higher doses or individual components alone may have adverse effects on cardiac function.

### Biochemical estimations in serum

**Figure 7.**
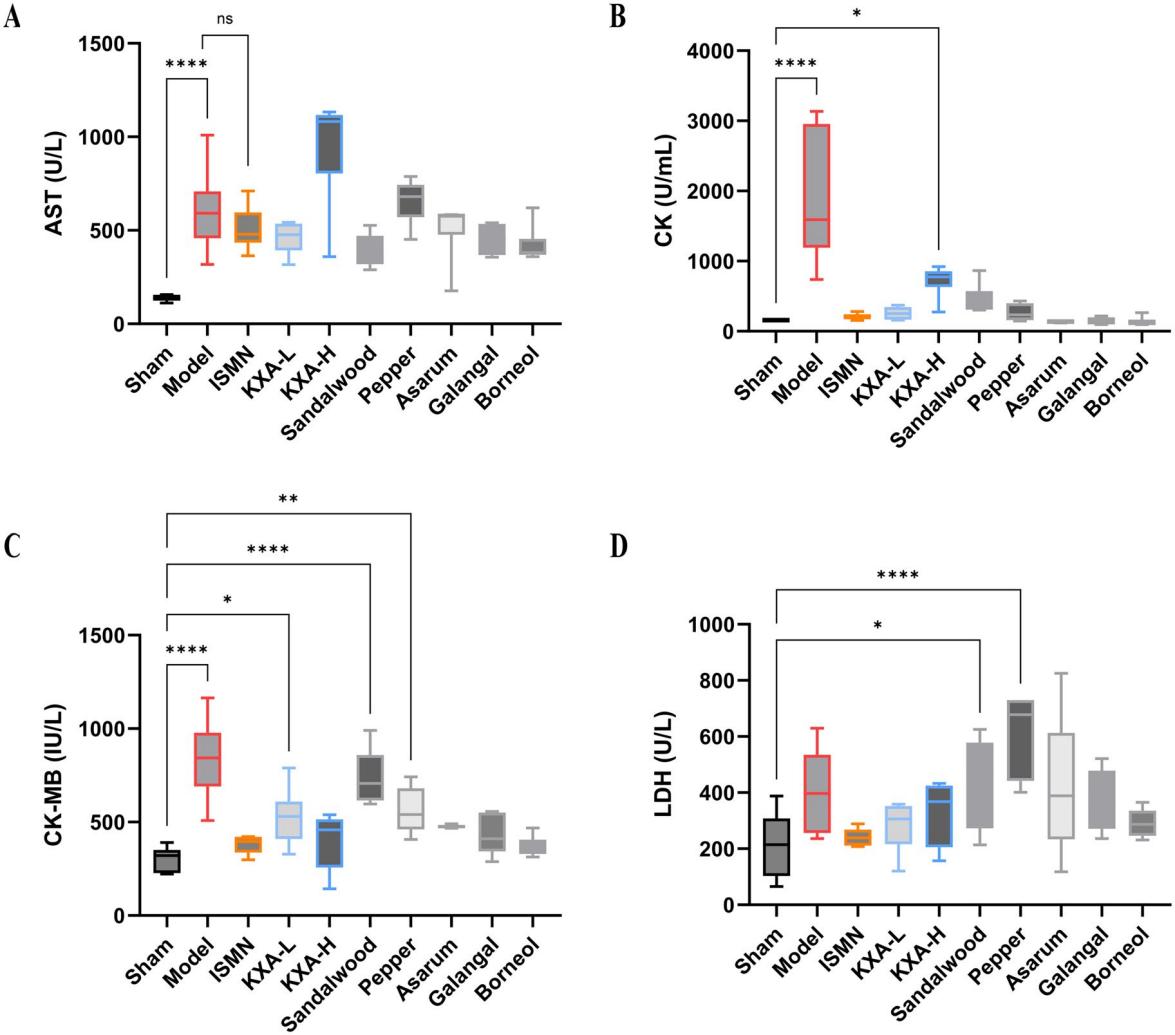
KXA-L improves serum markers of myocardial injury in AMI rats. Serum biochemical parameters, including aspartate aminotransferase (AST, A), creatine kinase (CK, B), creatine kinase-MB (CK-MB, C), and lactate dehydrogenase (LDH, D), in AMI rats treated with different doses of KXA and its individual herbal components. The data are presented as the mean ± SD (*n* = 6 per group). *p < 0.05 vs. sham, **p < 0.01 vs. sham, ***p < 0.001 vs. sham, ****p < 0.0001 vs. sham.

Serum biochemical assays revealed that at 24 h post-AMI induction, the model group showed significantly elevated levels of AST, CK, CK-MB, and LDH compared to sham controls, demonstrating the effective establishment of the myocardial injury model (Figure 7). Despite various drug interventions, none of the treatment groups showed a significant reduction in serum AST levels, indicating that these agents had limited effects on AST reduction (Figure 7A). Both the ISMN and KXA-L groups exhibited a significant decrease in CK, with the KXA-L group being more effective than the KXA-H group. Asarum oil, galangal oil, and borneol also lowered CK levels, with their efficacies slightly surpassing those of sandalwood and pepper oils (Figure 7B). KXA-L significantly reduced CK-MB levels, although these levels remained higher than those in the sham group. KXA-H showed a slight advantage over KXA-L. Sandalwood oil was ineffective in decreasing CK-MB levels, whereas pepper oil showed a minor improvement over sandalwood oil. Asarum oil, galangal oil, and borneol significantly reduced CK-MB levels (Figure 7C). Neither sandalwood oil nor pepper oil reduced LDH levels. In contrast, ISMN, both KXA dose groups, and the asarum oil, galangal oil, and borneol groups successfully restored LDH levels to normal (Figure 7D).

In summary, most interventions were effective in reducing CK, CK-MB, and LDH levels (except sandalwood oil and pepper oil for LDH). However, none of the agents produced a notable reduction in AST levels, reflecting both the beneficial and limited aspects of biochemical modulation by these treatments in acute myocardial injury.

## Discussion

Our comprehensive GC-MS profiling revealed that the volatile oils of sandalwood, pepper, galangal, and asarum, as well as borneol, possessed distinct chemical signatures and bioactive constituents. This study, designed to address the lack of systematic comparison between KXA and its individual components. The findings showed that these oils did not act as independent agents in an acute myocardial infarction (AMI) model. Instead, they exhibited complementary and, in some instances, compensatory actions, thereby exemplifying the fundamental concept of multi-component synergy that underpins many traditional medicinal formulations. Specifically, the KXA-L formulation consistently outperformed not only the individual components but also the high-dose formulation across multiple endpoints, including electrocardiographic recovery, infarct size reduction, hemodynamic stability, and histopathological improvement.

Sandalwood oil, rich in α-santalol and β-santalol, demonstrated robust antioxidant and anti-inflammatory activities, aligning with earlier reports of its tissue-protective potential in ischemia–reperfusion contexts (Misra and Dey 2013; Bommareddy et al. 2019). These properties likely contributed to its pronounced efficacy in mitigating ST-segment elevation and reducing infarct size in our AMI model, positioning it as a primary active ingredient within the KXA formula. Nevertheless, the findings from both the present study and previous investigations (Heuberger et al. 2006; Höferl et al. 2016) suggest that sandalwood oil can elevate systemic heart rate and ventricular preload, particularly at higher doses, potentially worsening myocardial fibrosis. This contrasting effect may be due to the pharmacokinetic and tissue-stability limitations of santalol, which might reduce its sustained inhibition of fibroblast activation and collagen synthesis. While ST-segment changes and infarct size reflect acute electrophysiological and perfusion improvements, anti-fibrotic effects represent structural remodeling processes that occur later and through different mechanisms. This dual profile underscores the need for careful dose titration and combination with other agents to maximize its benefits, while avoiding hemodynamic compromise.

Pepper oil, dominated by sesquiterpene compounds such as caryophyllene and humulene, is known for its cardioprotective effects. β-caryophyllene, for instance, has been shown to have cardioprotective effects *via* the modulation of inflammatory signaling pathways (Younis and Mohamed 2019; Meeran et al. 2021; Li et al. 2025; Chen et al. 2025b). In our study, however, pepper oil was less efficacious in providing acute protection, as measured by ST-segment and infarct size. Its key role instead appeared to be improving left ventricular compliance and contractility–functions essential for recovery after ischemia. This finding suggests a supportive role for pepper oil within a broader cardioprotective network when combined with strong anti-ischemic agents.

Asarum oil contains safrole and other phenylpropanoid derivatives known for their potent anti-inflammatory actions, notably through COX-1 modulation, which supports its benefits in ameliorating ischemic injury and improving certain serum markers (Liu et al. 2023; Happy et al. 2025). In the acute phase of AMI, safrole in asarum oil may provide transient benefits through anti-inflammatory and vasomodulatory mechanisms, such as the attenuation of ST-segment deviation. However, its high volatility, short half-life, and narrow therapeutic margin make it more suitable at low doses within multi-component formulations. This pharmacological profile offers a plausible explanation for the paradoxical finding that KXA-H is less effective than the low-dose regimen, as excessive safrole exposure may disrupt the delicate synergy of the formulation and and increase toxicity. Consistently, asarum oil alone exhibited a trend toward impaired ventricular relaxation, plausibly linked to the recognized toxicity profile of safrole, reinforcing the importance of strict proportion control in combination therapies.

Galangal oil, characterized by high levels of eucalyptol and monoterpenes, such as α-terpineol and D-limonene, exhibits both anti-inflammatory and cardioprotective properties (Paulino et al. 2022; Yang et al. 2023). These effects, while not always translating to pronounced reductions in acute ischemic indices in this study, may be attributable to the rapid plasma clearance and short elimination half-life of the principal constituent eucalyptol (Wu et al. 2020), resulting in insufficient tissue exposure. Monoterpenes can enhance endothelial NO signaling and exhibit vasorelaxant properties, improving ventricular relaxation and preload indices (left ventricular end-diastolic pressure, LVEDP; minimum rate of pressure decline, Min dP/dt) without necessarily increasing contractile pressure (Quintans et al. 2019). These vascular effects complement the stronger anti-ischemic agents, improving serum biomarkers and reducing myocardial fibrosis–benefits that the primary anti-ischemic components did not achieve alone.

Borneol is a bicyclic monoterpene widely used in traditional medicine and is often used as a penetration enhancer in formulations (Zhang et al. 2017; Kulkarni et al. 2021). In formulations, such as Compound Danshen Dropping Pills (Fufang Danshen Diwan) and Suoxiao Jiuxin Pills, borneol functions as an adjuvant to guide the therapeutic action to specific meridians and enhance efficacy. Pharmacokinetic–pharmacodynamic studies have demonstrated that the dynamic changes in plasma borneol concentrations at various time points following the administration of Danshen dropping pills are significantly correlated with variations in specific observed pharmacodynamic indicators (Zheng et al. 2022). In the present study, borneol did not markedly reduce acute ischemic indices, but it improved diastolic and contractile functions, restored ventricular compliance, and mitigated structural tissue alterations. These effects align with its traditional role as a harmonizing and balancing agent within the formula.

Collectively, these five components constitute an interdependent pharmacological network. Strong anti-ischemic agents (Sandalwood and Asarum) combined with hemodynamic stabilizers (Pepper), fibrosis modulators (Galangal), and functional enhancers (Borneol) provide multi-target cardioprotection. The consistently better performance of KXA-L across ECG, histopathology, hemodynamic, and biochemical measures supports the idea that efficacy arises from synergistic interactions rather than additive effects. In contrast, the comparatively attenuated efficacy observed with the higher dose (KXA-H) or with single-component administration may indicate a disruption of this pharmacological balance, raising the possibility of a non-linear, potentially inverted U-shaped dose–response relationship. Such non-linear dose–response patterns have been reported in pharmacological and toxicological studies, particularly for multi-component herbal formulations, and may reflect complex interactions among active constituents or counter-regulatory mechanisms at higher concentrations (Williamson 2001). However, this observation remains preliminary and warrants further investigation. From a translational perspective, these findings strongly support the precise formulation of multi-component herbal therapies for the treatment of AMI. Pathological processes, such as ischemia, ventricular fibrosis, inflammation, and altered hemodynamics, occur simultaneously in AMI, and successful intervention requires targeting each process in a coordinated fashion. By systematically comparing the whole formula to its parts, this study provides direct experimental evidence for the scientific rationale behind KXA’s composition, demonstrating that a purposefully balanced combination of volatile oils at an optimized dose provides broader and more stable cardioprotection than any individual constituent.

Despite its significant findings, this study has several limitations that should be acknowledged. The use of an acute rat model of myocardial infarction means the findings may not fully translate to the chronic progression of ischemic heart disease in humans. While our hemodynamic measurements provide valuable insights, future studies incorporating noninvasive imaging such as echocardiography would offer a more comprehensive evaluation of cardiac structural and functional recovery. Furthermore, while we documented the functional and biochemical outcomes, the underlying specific signaling pathways related to inflammation, fibrosis, and apoptosis to clarify how KXA achieves its multi-target cardioprotective effects should be explored. Additionally, the oral administration route used here for experimental consistency may differ in its pharmacokinetic profile from the clinical aerosol application of KXA. Nevertheless, these limitations do not diminish the study’s core contribution, which provides the first direct experimental validation of KXA’s synergistic design. Future research should be extended to chronic disease models and settings that more closely mimic clinical use, employ molecular pathway analysis, and address long-term safety to fully leverage the clinical therapeutic potential of synergistic botanical interventions. These results not only provide experimental evidence supporting the traditional rationale of KXA but also offer practical guidance for the rational design, dose optimization, and translational development of multi-component botanical therapies for acute myocardial infarction.

## Conclusions

Addressing a critical gap in the scientific validation of traditional multi-herbal therapies, this study was designed to systematically evaluate the cardioprotective effects of the complete KXA formulation against its individual components. Our findings conclusively demonstrate that KXA-L (120 µL/kg) provides significant multifaceted protection against acute myocardial ischemic injury in a preclinical model, outperforming both KXA-H (200 µL/kg) and single-agent interventions across a range of electrocardiographic, hemodynamic, biochemical, and histological endpoints. This work provides the first direct experimental evidence for the scientific rationale behind KXA’s traditional composition.

The significance of this study lies in its validation of a synergistic formulation, where the therapeutic outcome is greater and safer than the sum of its individual components. The potent anti-ischemic effects, driven primarily by sandalwood and asarum oils, were effectively complemented by the anti-fibrotic, anti-inflammatory, and functional support from galangal oil, pepper oil, and borneol. Crucially, the balanced combination mitigated the adverse effects observed with some individual components, such as increased myocardial fibrosis or impaired ventricular function.

These results provide the foundation for future investigations aimed at maximizing KXA’s clinical potential. Future studies should focus on elucidating the underlying molecular mechanisms of this synergy and extend these findings to chronic models of ischemic heart disease. Furthermore, optimizing component ratios and evaluating long-term safety and efficacy are essential next steps for translating this promising traditional medicine into a validated, evidence-based therapy in modern cardiovascular care. Ultimately, these insights pave the way for the potential integration of KXA into clinical practice as a novel adjunctive therapy for ischemic heart disease.

## Supplementary Material

Supplementary Tables S1 to S4.docx

## Data Availability

The data that support the findings of this research are available from the corresponding author upon reasonable request.
