# Supplementary material for: Comparative cardioprotective effects of Kuanxiong Aerosol and its individual components in a rat model of acute myocardial infarction
Source: Pharm Biol. 2026 Feb 15;64(1):295–311. doi: 10.1080/13880209.2026.2626099 (PMC12912229; doi:10.1080/13880209.2026.2626099)
Supplement: Supplementary Tables S1 to S4.docx [file IPHB_A_2626099_SM8315.docx]

**Supplementary Tables S1–S4 with captions.**

**Table S1.** Identification of major components of Sandalwood oil

| **No.** | **RT** | **Compound Name** | **CAS#** | **Formula** | **Area MI** | **Match Score** | **Area%-T** | **Area%-M** |
| --- | --- | --- | --- | --- | --- | --- | --- | --- |
| 1 | 7.2457 | Benzene, 1,3-dimethyl | 108-38-3 | C_8_H_10_ | 201177 | 96.5 | 0.03 | 0.14 |
| 2 | 18.3225 | Spiro[2.4]heptane, 1,5-dimethyl-6-methylene | 62238-24-8 | C_10_H_16_ | 597950 | 81.7 | 0.1 | 0.4 |
| 3 | 18.3225 | .alpha.-Santalol | 115-71-9 | C_15_H_24_O | 601386 | 81.6 | 0.1 | 0.41 |
| 4 | 18.7094 | Tricyclo[7.1.0.0[1,3]]decane-2-carbaldehyde | 1000305-39-2 | C_11_H_16_O | 171440 | 77.8 | 0.03 | 0.12 |
| 5 | 18.7421 | Bicyclo[2.2.1]heptane, 2-methyl-3-methylene-2-(4-methyl-3-pentenyl)-, (1S-exo) | 511-59-1 | C_15_H_24_ | 161727 | 73.7 | 0.03 | 0.11 |
| 6 | 19.2603 | Bicyclo[2.2.1]heptane, 2-methyl-3-methylene-2-(4-methyl-3-pentenyl)-, (1S-exo) | 511-59-1 | C_15_H_24_ | 254185 | 89.5 | 0.04 | 0.17 |
| 7 | 19.2774 | Bicyclo[2.2.1]hept-2-ene, 2,3-dimethyl | 529-16-8 | C_9_H_14_ | 272800 | 89.4 | 0.04 | 0.18 |
| 8 | 19.6107 | 2,3-Dimethyltricyclo[2.2.1.02,6]heptane-3-carboxylic acid | 562-66-3 | C_10_H_14_O_2_ | 173572 | 89 | 0.03 | 0.12 |
| 9 | 21.5098 | 3-((3R)-2,3-Dimethyltricyclo[2.2.1.02,6]heptan-3yl)propanal | 16933-18-9 | C_12_H_18_O | 3666739 | 99.4 | 0.59 | 2.47 |
| 10 | 21.5969 | 3-Octene-2,6-dione, 5,5,7-trimethyl-, (E) | 77142-73-5 | C_11_H_18_O_2_ | 179951 | 72.5 | 0.03 | 0.12 |
| 11 | 21.8816 | 3-((1S,5S,6R)-2,6-Dimethylbicyclo[3.1.1]hept-2-en-6yl)propanal | 203499-08-5 | C_12_H_18_O | 728091 | 92.2 | 0.12 | 0.49 |
| 12 | 22.3935 | Tricyclo[4.3.1.1<2,5>]undec-3-en-10-ol, 10-methyl-, stereoisomer | 70220-96-1 | C_12_H_18_O | 1550682 | 86.4 | 0.25 | 1.05 |
| 13 | 22.6985 | 2,5-Furandicarboxaldehyde | 823-82-5 | C_6_H_4_O_3_ | 166651 | 79.2 | 0.03 | 0.11 |
| 14 | 22.699 | .alpha.-Farnesene | 502-61-4 | C_15_H_24_ | 231594 | 75.9 | 0.04 | 0.16 |
| 15 | 22.8599 | (1S,5S)-2-Methyl-5-((R)-6-methylhept-5-en-2yl)bicyclo[3.1.0]hex-2-ene | 159407-35-9 | C_15_H_24_ | 298091 | 93 | 0.05 | 0.2 |
| 16 | 23.03 | Petasitene | 443124-67-2 | C_15_H_24_ | 269128 | 91.4 | 0.04 | 0.18 |
| 17 | 23.2048 | 2-Epi-.alpha.-funebrene | 65354-33-8 | C_15_H_24_ | 442985 | 88.8 | 0.07 | 0.3 |
| 18 | 23.4835 | 1H-3a,7-Methanoazulene, 2,3,4,7,8,8a-hexahydro3,6,8,8-tetramethyl-, [3R(3.alpha.,3a.beta.,7.beta.,8a.alpha.)] | 469-61-4 | C_15_H_24_ | 797081 | 95.6 | 0.13 | 0.54 |
| 19 | 23.6467 | Tricyclo[2.2.1.0(2,6)]heptane, 1,7-dimethyl-7-(4-methyl-3-pentenyl)-, (-) | 512-61-8 | C_15_H_24_ | 24314997 | 99.4 | 3.93 | 16.4 |
| 20 | 23.7512 | 6,8-Nonadien-2-one, 6-methyl-5-(1-methylethylidene)- | 60714-16-1 | C_13_H_20_O | 289535 | 79.5 | 0.05 | 0.2 |
| 21 | 24.0341 | cis-.alpha.-Bergamotene | 18252-46-5 | C_15_H_24_ | 5509590 | 98.1 | 0.89 | 3.72 |
| 22 | 24.3445 | Bicyclo[2.2.1]heptane, 2-methyl-3-methylene-2-(4-methyl-3-pentenyl)-, (1S-endo) | 25532-78-9 | C_15_H_24_ | 27925138 | 99.3 | 4.51 | 18.83 |
| 23 | 24.5645 | Benzenemethanol, 4-methyl-.alpha.-(1-methyl-2propenyl)-, (R*,R*) | 83173-76-6 | C_12_H_16_O | 284141 | 73.5 | 0.05 | 0.19 |
| 24 | 24.6668 | Bicyclo[2.2.1]heptane, 2-methyl-3-methylene-2-(4-methyl-3-pentenyl)-, (1S-endo) | 25532-78-9 | C_15_H_24_ | 41088322 | 98.4 | 6.63 | 27.71 |
| 25 | 24.8426 | Acoradiene,beta | 28477-64-7 | C_15_H_24_ | 1368885 | 91.5 | 0.22 | 0.92 |
| 26 | 25.1229 | 1-Methyl-4-(6-methylhept-5-en-2-yl)cyclohexa-1,3diene | 451-55-8 | C_15_H_24_ | 1570976 | 98 | 0.25 | 1.06 |
| 27 | 25.2046 | Benzene, 1-(1,5-dimethyl-4-hexenyl)-4-methyl | 644-30-4 | C_15_H_22_ | 9765372 | 99 | 1.58 | 6.58 |
| 28 | 25.277 | (1S,5S,6R)-6-Methyl-2-methylene-6-(4-methylpent-3en-1-yl)bicyclo[3.1.1]heptane | 15438-94-5 | C_15_H_24_ | 776784 | 85.4 | 0.13 | 0.52 |
| 29 | 25.4021 | Helifolen-12-al B (anti-anti-anti-) | 235095-63-3 | C_15_H_22_O | 797002 | 86.6 | 0.13 | 0.54 |
| 30 | 25.4762 | (R)-1-Methyl-4-(6-methylhept-5-en-2-yl)cyclohexa1,4-diene | 28976-67-2 | C_15_H_24_ | 451541 | 82.8 | 0.07 | 0.3 |
| 31 | 25.4767 | (R,Z)-2-Methyl-6-(4-methylcyclohexa-1,4-dien-1yl)hept-2-en-1-ol | 698365-10-5 | C_15_H_24_O | 498448 | 82.8 | 0.08 | 0.34 |
| 32 | 25.8378 | .beta.-Bisabolene | 495-61-4 | C_15_H_24_ | 2220830 | 94.1 | 0.36 | 1.5 |
| 33 | 25.913 | (R)-1-Methyl-4-(6-methylhept-5-en-2-yl)cyclohexa1,4-diene | 28976-67-2 | C_15_H_24_ | 5813305 | 95.1 | 0.94 | 3.92 |
| 34 | 25.9353 | Butylated Hydroxytoluene | 128-37-0 | C_15_H_24_O | 774231 | 84.9 | 0.12 | 0.52 |
| 35 | 25.9707 | 5-(2,3-Dimethyltricyclo[2.2.1.02,6]heptan-3-yl)pentan2-one | 60843-83-6 | C_14_H_22_O | 1188700 | 75.4 | 0.19 | 0.8 |
| 36 | 26.0519 | 5-(2,3-Dimethyltricyclo[2.2.1.02,6]heptan-3-yl)pentan2-one | 60843-83-6 | C_14_H_22_O | 1463716 | 89.3 | 0.24 | 0.99 |
| 37 | 26.1948 | Benzene, (1-methoxy-4-methyl-3-pentenyl) | 68705-86-2 | C_13_H_18_O | 2500393 | 83.1 | 0.4 | 1.69 |
| 38 | 26.1982 | .alpha.-Santalol | 115-71-9 | C_15_H_24_O | 2907060 | 83.5 | 0.47 | 1.96 |
| 39 | 26.3887 | (E)-1-Methyl-4-(6-methylhept-5-en-2ylidene)cyclohex-1-ene | 53585-13-0 | C_15_H_24_ | 250668 | 79.2 | 0.04 | 0.17 |
| 40 | 26.652 | Cyclohexene, 4-[(1E)-1,5-dimethyl-1,4-hexadien-1-yl]1-methyl | 25532-79-0 | C_15_H_24_ | 334669 | 74.5 | 0.05 | 0.23 |
| 41 | 26.7344 | .alpha.-Santalol | 115-71-9 | C_15_H_24_O | 1773667 | 81 | 0.29 | 1.2 |
| 42 | 26.7633 | Tricyclo[2.2.1.0(2,6)]heptane-3-methanol, 2,3dimethyl | 29550-55-8 | C_10_H_16_O | 4157457 | 78.6 | 0.67 | 2.8 |
| 43 | 27.0546 | Tricyclo[2.2.1.0(2,6)]heptane-3-methanol, 2,3dimethyl | 29550-55-8 | C_10_H_16_O | 1456046 | 79.5 | 0.24 | 0.98 |
| 44 | 27.1579 | Tricyclo[3.1.0.0(2,4)]hexane, 3,3,6,6-tetramethyl-, (1.alpha.,2.beta.,4.beta.,5.alpha.) | 58987-01-2 | C_10_H_16_ | 4013488 | 85.9 | 0.65 | 2.71 |
| 45 | 27.2549 | 1-((1S,3aR,4S,7R,7aR)-3a,7a-Dimethyloctahydro-1H4,7-methanoinden-1-yl)ethanone | 66748-84-3 | C_14_H_22_O | 471568 | 71.2 | 0.08 | 0.32 |
| 46 | 27.2561 | 1,4-Methano-1H-indene, octahydro-1,7a-dimethyl-4(1-methylethenyl)-, [1S(1.alpha.,3a.beta.,4.alpha.,7a.beta.)] | 87064-18-4 | C_15_H_24_ | 425099 | 71.1 | 0.07 | 0.29 |
| 47 | 27.3593 | Furan, 3-(4,8-dimethyl-3,7-nonadienyl)-, (E) | 23262-34-2 | C_15_H_22_O | 762016 | 82.3 | 0.12 | 0.51 |
| 48 | 27.4287 | Benzenemethanol, 4-methyl-.alpha.-(1-methyl-2propenyl)-, (R*,R*) | 83173-76-6 | C_12_H_16_O | 506621 | 80.5 | 0.08 | 0.34 |
| 49 | 27.6506 | 1-((1S,3aR,4S,7R,7aR)-3a,7a-Dimethyloctahydro-1H4,7-methanoinden-1-yl)ethanone | 66748-84-3 | C_14_H_22_O | 4843544 | 98.7 | 0.78 | 3.27 |
| 50 | 27.874 | Humulene | 6753-98-6 | C_15_H_24_ | 2653257 | 82 | 0.43 | 1.79 |
| 51 | 27.8915 | (1S,2E,6E,10R)-3,7,11,11Tetramethylbicyclo[8.1.0]undeca-2,6-diene | 24703-35-3 | C_15_H_24_ | 1915313 | 82 | 0.31 | 1.29 |
| 52 | 28.0629 | .beta.-Santalol | 77-42-9 | C_15_H_24_O | 1081730 | 76.1 | 0.17 | 0.73 |
| 53 | 28.0637 | Bicyclo[4.1.0]heptan-2-one, 3,4,4-trimethyl-3-(3methyl-1,3-butadienyl)-, [1.alpha.,3.alpha.(E),6.alpha.]-(.+-.) | 102146-81-6 | C_15_H_22_O | 1000119 | 70.5 | 0.16 | 0.67 |
| 54 | 28.1741 | 1-Cyclopentene-1-methanol, 2-methyl-5-(1methylethyl) | 80113-82-2 | C_10_H_18_O | 474334 | 75.9 | 0.08 | 0.32 |
| 55 | 28.3604 | 6-Isopropenyl-4,8a-dimethyl-4a,5,6,7,8,8a-hexahydro1H-naphthalen-2-one | 86917-79-5 | C_15_H_22_O | 1376997 | 85.9 | 0.22 | 0.93 |
| 56 | 28.3605 | 7-Oxabicyclo[4.1.0]heptane, 2,2,6-trimethyl-1-(3methyl-1,3-butadienyl)-5-methylene | 70038-20-9 | C_15_H_22_O | 1417819 | 85.7 | 0.23 | 0.96 |
| 57 | 28.6274 | 3,8,8-Trimethyl-4-oxatricyclo[5.1.0.0,3,5]octane | 2225-98-1 | C_10_H_16_O | 847255 | 77.9 | 0.14 | 0.57 |
| 58 | 28.6287 | Cedroxyde | 13786-79-3 | C_15_H_24_O | 945208 | 78.4 | 0.15 | 0.64 |
| 59 | 28.8956 | Cyclopentanone, 2,5-dicyclopentylidene | 5682-82-6 | C_15_H_20_O | 2451979 | 86 | 0.4 | 1.65 |
| 60 | 29.1262 | 2-((2R,8R,8aS)-8,8a-Dimethyl-1,2,3,4,6,7,8,8aoctahydronaphthalen-2-yl)propan-2-ol | 20489-45-6 | C_15_H_26_O | 775648 | 75.2 | 0.13 | 0.52 |
| 61 | 29.1377 | Germacrene D | 23986-74-5 | C_15_H_24_ | 904065 | 72.1 | 0.15 | 0.61 |
| 62 | 29.3088 | Cyclobuta[1,2:3,4]dicyclooctene, 1,2,5,6,6a,6b,7,8,11,12,12a,12b-dodecahydro-, (6a.alpha.,6b.alpha.,12a.beta.,12b.beta.) | 61233-68-9 | C_16_H_24_ | 21345515 | 84.8 | 3.45 | 14.39 |
| 63 | 29.5481 | (E)-5-((1R,3R,6S)-2,3Dimethyltricyclo[2.2.1.02,6]heptan-3-yl)-2methylpent-2-enal | 19903-70-9 | C_15_H_22_O | 1235880 | 81.7 | 0.2 | 0.83 |
| 64 | 29.608 | Benzo[b]thiophene, 2-ethyl | 1196-81-2 | C_10_H_10_S | 345186 | 79.6 | 0.06 | 0.23 |
| 65 | 29.6507 | 3-Cyclohexen-1-ol, 1-[(1S)-1,5-dimethyl-4-hexenyl]-4methyl-, (1S) | 15352-77-9 | C_15_H_26_O | 15935897 | 91.2 | 2.57 | 10.75 |
| 66 | 29.7397 | 4-[(E)-5-Hydroxy-3-methylpent-3-enyl]-3,5,5trimethylcyclohex-2-en-1-one | 1083195-46-3 | C_15_H_24_O_2_ | 13187822 | 78.4 | 2.13 | 8.89 |
| 67 | 29.831 | (E)-5-((1R,3R,6S)-2,3Dimethyltricyclo[2.2.1.02,6]heptan-3-yl)-2methylpent-2-enal | 19903-70-9 | C_15_H_22_O | 52241706 | 93.2 | 8.43 | 35.23 |
| 68 | 29.9083 | 3,5-Dimethyl-1-dimethylisopropylsilyloxybenzene | 1000307-91-0 | C_13_H_22_OSi | 790327 | 81.3 | 0.13 | 0.53 |
| 69 | 29.9409 | .alpha.-Santalol | 115-71-9 | C_15_H_24_O | 148304113 | 99.2 | 23.94 | 100 |
| 70 | 30.4435 | (Z)-epi-.beta.-Santalol | 79081-90-6 | C_15_H_24_O | 11963545 | 86 | 1.93 | 8.07 |
| 71 | 30.4443 | .beta.-Santalol | 77-42-9 | C_15_H_24_O | 12388823 | 87.3 | 2 | 8.35 |
| 72 | 30.5593 | .alpha.-Santalyl formate | 1345675-64-0 | C_16_H_24_O_2_ | 18620134 | 86.6 | 3.01 | 12.56 |
| 73 | 30.5594 | (+)-Epi-.beta.-santalyl acetate | 41414-75-9 | C_17_H_26_O_2_ | 27898968 | 83.2 | 4.5 | 18.81 |
| 74 | 30.7608 | .beta.-Santalol | 77-42-9 | C_15_H_24_O | 44416160 | 85.9 | 7.17 | 29.95 |
| 75 | 30.7693 | Pyrazole-3-carbohydrazide, 4-bromo-1-methyl | 1000273-84-9 | C_5_H_7_BrN_4_O | 481954 | 75.1 | 0.08 | 0.32 |
| 76 | 30.8143 | l-Leucine, N-methyl-N-(2-methoxyethoxycarbonyl)-, tetradecyl ester | 1000328-54-8 | C_25_H_49_NO_5_ | 234107 | 83.8 | 0.04 | 0.16 |
| 77 | 31.3008 | 1,2-Benzenediol, O-(4-methylbenzoyl)-O'propoxycarbonyl | 1010329-75-8 | C_18_H_18_O_5_ | 27444429 | 73.6 | 4.43 | 18.51 |
| 78 | 31.4174 | .beta.-Santalol | 77-42-9 | C_15_H_24_O | 29200415 | 77.9 | 4.71 | 19.69 |
| 79 | 31.4217 | Bicyclo[2.2.1]hept-2-ene, 2,3-dimethyl | 529-16-8 | C_9_H_14_ | 12220809 | 79.9 | 1.97 | 8.24 |
| 80 | 33.7362 | 2-Pyrrolidinone, 5-(cyclohexylmethyl) | 14293-08-4 | C_11_H_19_NO | 3147902 | 72.6 | 0.51 | 2.12 |
| 81 | 35.5913 | Dibutyl phthalate | 84-74-2 | C_16_H_22_O_4_ | 623891 | 95.6 | 0.1 | 0.42 |
| 82 | 48.7461 | .alpha.-Santalyl formate | 1345675-64-0 | C_16_H_24_O_2_ | 299361 | 76.1 | 0.05 | 0.2 |

**Table S2.** Identification of major components of Pepper oil

| **No.** | **RT** | **Compound Name** | **CAS#** | **Formula** | **Area MI** | **Match Score** | **Area%-T** | **Area%-M** |
| --- | --- | --- | --- | --- | --- | --- | --- | --- |
| 1 | 9.2725 | α-Pinene | 80-56-8 | C_10_H_16_ | 4625426 | 99.2 | 0.19 | 2.02 |
| 2 | 9.7404 | Camphene | 79-92-5 | C_10_H_16_ | 435013 | 96.7 | 0.02 | 0.19 |
| 3 | 10.6557 | Bicyclo[3.1.1]heptane, 6,6-dimethyl-2-methylene-, (1S) | 18172-67-3 | C_10_H_16_ | 3425977 | 98.8 | 0.14 | 1.49 |
| 4 | 11.1878 | β-Myrcene | 123-35-3 | C_10_H_16_ | 525076 | 96.5 | 0.02 | 0.23 |
| 5 | 11.7538 | Tricyclo[2.2.1.0(2,6)]heptane, 1,3,3-trimethyl | 488-97-1 | C_10_H_16_ | 570244 | 97.4 | 0.02 | 0.25 |
| 6 | 12.3574 | D-Limonene | 5989-27-5 | C_10_H_16_ | 2227428 | 98.6 | 0.09 | 0.97 |
| 7 | 12.4342 | Eucalyptol | 470-82-6 | C_10_H_18_O | 601162 | 97.9 | 0.03 | 0.26 |
| 8 | 12.6962 | trans-β-Ocimene | 3779-61-1 | C_10_H_16_ | 1777709 | 99.2 | 0.07 | 0.77 |
| 9 | 13.0182 | β-Ocimene | 13877-91-3 | C_10_H_16_ | 2021757 | 98.9 | 0.08 | 0.88 |
| 10 | 14.3931 | 2-Nonanone | 821-55-6 | C_9_H_18_O | 463085 | 95.9 | 0.02 | 0.2 |
| 11 | 14.6298 | Linalool | 78-70-6 | C_10_H_18_O | 18619804 | 96.4 | 0.78 | 8.11 |
| 12 | 15.9405 | Camphor | 76-22-2 | C_10_H_16_O | 233171 | 86.9 | 0.01 | 0.1 |
| 13 | 17.6069 | Dodecane | 112-40-3 | C_12_H_26_ | 276000 | 81.7 | 0.01 | 0.12 |
| 14 | 19.7854 | Benzenepropanoic acid, methyl ester | 103-25-3 | C_10_H_12_O_2_ | 408497 | 90.4 | 0.02 | 0.18 |
| 15 | 20.0878 | 6-Tridecene, (E) | 6434-76-0 | C_13_H_26_ | 10042740 | 97.5 | 0.42 | 4.38 |
| 16 | 20.2627 | 2-Undecanone | 112-12-9 | C_11_H_22_O | 787104 | 97.1 | 0.03 | 0.34 |
| 17 | 20.4337 | Tridecane | 629-50-5 | C_13_H_28_ | 64455848 | 99.5 | 2.69 | 28.09 |
| 18 | 21.4883 | Cyclohexene, 4-ethenyl-4-methyl-3-(1-methylethenyl)-1-(1-methylethyl)-, (3R-trans) | 20307-84-0 | C_15_H_24_ | 2843910 | 98.1 | 0.12 | 1.24 |
| 19 | 21.8115 | α-Cubebene | 17699-14-8 | C_15_H_24_ | 2116107 | 98.5 | 0.09 | 0.92 |
| 20 | 22.2075 | 1-(1-Ethyl-2,3-dimethyl-cyclopent-2-enyl)-ethanone | 1000185-18-6 | C_11_H_18_O | 796627 | 90 | 0.03 | 0.35 |
| 21 | 22.3001 | 1,2,4-Metheno-1H-indene, octahydro-1,7a-dimethyl-5(1-methylethyl)-, [1S(1.alpha.,2.alpha.,3a.beta.,4.alpha.,5.alpha.,7a.beta.,8 S*)] | 22469-52-9 | C_15_H_24_ | 412814 | 86.8 | 0.02 | 0.18 |
| 22 | 22.3015 | (1R,4aS,8aR)-1-Isopropyl-4,7-dimethyl-1,2,4a,5,6,8ahexahydronaphthalene | 20085-19-2 | C_15_H_24_ | 403425 | 86.2 | 0.02 | 0.18 |
| 23 | 22.4006 | Ylangene | 14912-44-8 | C_15_H_24_ | 1009720 | 96 | 0.04 | 0.44 |
| 24 | 22.5208 | Copaene | 3856-25-5 | C_15_H_24_ | 10461794 | 98.1 | 0.44 | 4.56 |
| 25 | 22.7539 | Prenyl limonene,trans | 135267-19-5 | C_15_H_24_ | 963530 | 73.3 | 0.04 | 0.42 |
| 26 | 22.7572 | (-)-β-Bourbonene | 5208-59-3 | C_15_H_24_ | 1907583 | 92.3 | 0.08 | 0.83 |
| 27 | 22.8896 | D-Alanine, N-(4-butylbenzoyl)-, heptyl ester | 1000354-10-2 | C_21_H_33_NO_3_ | 601410 | 71.3 | 0.03 | 0.26 |
| 28 | 22.9397 | Cyclohexane, 1-ethenyl-1-methyl-2,4-bis(1methylethenyl)-, [1S-(1.alpha.,2.beta.,4.beta.)] | 515-13-9 | C_15_H_24_ | 30468743 | 98.8 | 1.27 | 13.28 |
| 29 | 23.0725 | Tetradecane | 629-59-4 | C_14_H_30_ | 541258 | 97.8 | 0.02 | 0.24 |
| 30 | 23.2733 | (1S,5S)-2-Methyl-5-((R)-6-methylhept-5-en-2yl)bicyclo[3.1.0]hex-2-ene | 159407-35-9 | C_15_H_24_ | 3421647 | 97.9 | 0.14 | 1.49 |
| 31 | 23.5311 | cis-α-Bergamotene | 18252-46-5 | C_15_H_24_ | 14491561 | 98.2 | 0.6 | 6.32 |
| 32 | 23.7008 | Caryophyllene | 87-44-5 | C_15_H_24_ | 229466888 | 99.8 | 9.58 | 100 |
| 33 | 23.9059 | (1R,2S,6S,7S,8S)-8-Isopropyl-1-methyl-3methylenetricyclo[4.4.0.02,7]decane-rel | 18252-44-3 | C_15_H_24_ | 1643080 | 98.3 | 0.07 | 0.72 |
| 34 | 24.0426 | cis-α-Bergamotene | 18252-46-5 | C_15_H_24_ | 23229028 | 98.2 | 0.97 | 10.12 |
| 35 | 24.2248 | Cyclohexene, 3-(1,5-dimethyl-4-hexenyl)-6methylene-, [S-(R*,S*)] | 20307-83-9 | C_15_H_24_ | 430752 | 90.6 | 0.02 | 0.19 |
| 36 | 24.2941 | Naphthalene, 1,2,3,4,4a,5,6,8a-octahydro-7-methyl-4methylene-1-(1-methylethyl)-, (1.alpha.,4a.beta.,8a.alpha.) | 39029-41-9 | C_15_H_24_ | 275940 | 83.1 | 0.01 | 0.12 |
| 37 | 24.2952 | 1H-3a,7-Methanoazulene, 2,3,4,7,8,8a-hexahydro3,6,8,8-tetramethyl-, [3R(3.alpha.,3a.beta.,7.beta.,8a.alpha.)] | 469-61-4 | C_15_H_24_ | 267090 | 78.3 | 0.01 | 0.12 |
| 38 | 24.3528 | Bicyclo[2.2.1]heptane, 2-methyl-3-methylene-2-(4methyl-3-pentenyl)-, (1S-endo) | 25532-78-9 | C_15_H_24_ | 709623 | 91 | 0.03 | 0.31 |
| 39 | 24.4487 | Alloaromadendrene | 25246-27-9 | C_15_H_24_ | 2091322 | 89.6 | 0.09 | 0.91 |
| 40 | 24.5479 | Humulene | 6753-98-6 | C_15_H_24_ | 173232689 | 97.7 | 7.23 | 75.49 |
| 41 | 24.5488 | (1S,2E,6E,10R)-3,7,11,11Tetramethylbicyclo[8.1.0]undeca-2,6-diene | 24703-35-3 | C_15_H_24_ | 121003934 | 80.6 | 5.05 | 52.73 |
| 42 | 24.6654 | Bicyclo[2.2.1]heptane, 2-methyl-3-methylene-2-(4methyl-3-pentenyl)-, (1S-exo) | 511-59-1 | C_15_H_24_ | 3278032 | 97.4 | 0.14 | 1.43 |
| 43 | 24.7637 | (+)-epi-Bicyclosesquiphellandrene | 54274-73-6 | C_15_H_24_ | 372507 | 79.3 | 0.02 | 0.16 |
| 44 | 24.764 | Bicyclosesquiphellandrene | 54324-03-7 | C_15_H_24_ | 331848 | 82.4 | 0.01 | 0.14 |
| 45 | 24.829 | 1,3-Methanopentalene, octahydro | 13913-22-9 | C_9_H_14_ | 311293 | 72.1 | 0.01 | 0.14 |
| 46 | 24.9184 | (4R,4aS,6S)-4,4a-Dimethyl-6-(prop-1-en-2-yl)1,2,3,4,4a,5,6,7-octahydronaphthalene | 823810-22-6 | C_15_H_24_ | 323245 | 78.9 | 0.01 | 0.14 |
| 47 | 25.0159 | aR-Himachalene | 19419-67-1 | C_15_H_22_ | 354400 | 75.4 | 0.01 | 0.15 |
| 48 | 25.0986 | N,N-bis(1-(p-tolyl)propan-2-yl)formamide | 1010456-37-0 | C_21_H_27_NO | 1338379 | 71.5 | 0.06 | 0.58 |
| 49 | 25.119 | 1-Pentadecene | 13360-61-7 | C_15_H_30_ | 76255596 | 96.6 | 3.18 | 33.23 |
| 50 | 25.2374 | Germacrene D | 23986-74-5 | C_15_H_24_ | 133893272 | 93.1 | 5.59 | 58.35 |
| 51 | 25.2729 | 1-Pentadecene | 13360-61-7 | C_15_H_30_ | 89514957 | 91.1 | 3.74 | 39.01 |
| 52 | 25.3645 | Naphthalene, decahydro-4a-methyl-1-methylene-7-(1methylethenyl)-, [4aR-(4a.alpha.,7.alpha.,8a.beta.)] | 17066-67-0 | C_15_H_24_ | 51923213 | 98.8 | 2.17 | 22.63 |
| 53 | 25.452 | Benzene, 1,3,5-tris(1-methylethyl) | 717-74-8 | C_15_H_24_ | 696102 | 80 | 0.03 | 0.3 |
| 54 | 25.5388 | 1,3-Cyclohexadiene, 5-(1,5-dimethyl-4-hexenyl)-2methyl-, [S-(R*,S*)] | 495-60-3 | C_15_H_24_ | 97495596 | 92 | 4.07 | 42.49 |
| 55 | 25.591 | 1,2-Bis(4-methoxyphenyl)ethane-1,2-diamine | 51208-43-6 | C_16_H_20_N_2_O_2_ | 223074 | 77.1 | 0.01 | 0.1 |
| 56 | 25.6235 | Pentadecane | 629-62-9 | C_15_H_32_ | 165919453 | 82.3 | 6.92 | 72.31 |
| 57 | 25.7072 | cis-α-Bisabolene | 29837-07-8 | C_15_H_24_ | 40105630 | 96.6 | 1.67 | 17.48 |
| 58 | 25.8788 | β-Bisabolene | 495-61-4 | C_15_H_24_ | 152274201 | 98.9 | 6.36 | 66.36 |
| 59 | 25.9362 | (R)-1-Methyl-4-(6-methylhept-5-en-2-yl)cyclohexa1,4-diene | 28976-67-2 | C_15_H_24_ | 4746544 | 96.2 | 0.2 | 2.07 |
| 60 | 25.9556 | Butylated Hydroxytoluene | 128-37-0 | C_15_H_24_O | 540196 | 81.8 | 0.02 | 0.24 |
| 61 | 26.0369 | Naphthalene, 1,2,3,4,4a,5,6,8a-octahydro-7-methyl-4methylene-1-(1-methylethyl)-, (1.alpha.,4a.beta.,8a.alpha.) | 39029-41-9 | C_15_H_24_ | 2836256 | 94.1 | 0.12 | 1.24 |
| 62 | 26.1328 | (-)-α-Panasinsen | 56633-28-4 | C_15_H_24_ | 43114723 | 97.7 | 1.8 | 18.79 |
| 63 | 26.227 | 1H-3a,7-Methanoazulene, octahydro-3,8,8-trimethyl6-methylene-, [3R(3.alpha.,3a.beta.,7.beta.,8a.alpha.)] | 546-28-1 | C_15_H_24_ | 27653848 | 97 | 1.15 | 12.05 |
| 64 | 26.2318 | Naphthalene, 1,2,3,5,6,8a-hexahydro-4,7-dimethyl-1(1-methylethyl)-, (1S-cis) | 483-76-1 | C_15_H_24_ | 8682283 | 71.8 | 0.36 | 3.78 |
| 65 | 26.4146 | (E)-1-Methyl-4-(6-methylhept-5-en-2ylidene)cyclohex-1-ene | 53585-13-0 | C_15_H_24_ | 25710643 | 98.3 | 1.07 | 11.2 |
| 66 | 26.6612 | Cyclohexene, 4-[(1E)-1,5-dimethyl-1,4-hexadien-1-yl]1-methyl | 25532-79-0 | C_15_H_24_ | 62579939 | 96.3 | 2.61 | 27.27 |
| 67 | 26.7122 | α-Calacorene | 21391-99-1 | C_15_H_20_ | 245321 | 83.2 | 0.01 | 0.11 |
| 68 | 26.9455 | Caryophyllene oxide | 1139-30-6 | C_15_H_24_O | 1628465 | 95.7 | 0.07 | 0.71 |
| 69 | 27.0582 | 1,5-Cyclodecadiene, 1,5-dimethyl-8-(1methylethylidene)-, (E,E) | 15423-57-1 | C_15_H_24_ | 1259212 | 97.1 | 0.05 | 0.55 |
| 70 | 27.1865 | β-Guaiene | 88-84-6 | C_15_H_24_ | 770621 | 70.5 | 0.03 | 0.34 |
| 71 | 27.2809 | (1S,3aR,4R,8R,8aS)-1-Isopropyl-3a-methyl-7methylenedecahydro-4,8-epoxyazulene | 88395-47-5 | C_15_H_24_O | 561889 | 81.1 | 0.02 | 0.24 |
| 72 | 27.6819 | Caryophyllene oxide | 1139-30-6 | C_15_H_24_O | 42391202 | 99 | 1.77 | 18.47 |
| 73 | 27.7795 | Flourensadiol | 55812-89-0 | C_15_H_26_O_2_ | 886277 | 77 | 0.04 | 0.39 |
| 74 | 27.7799 | Eremophila ketone | 158930-41-7 | C_15_H_24_O | 816908 | 72.8 | 0.03 | 0.36 |
| 75 | 27.9259 | Phytol | 150-86-7 | C_20_H_40_O | 1482678 | 73.4 | 0.06 | 0.65 |
| 76 | 28.0379 | Humulene epoxide I | 19888-33-6 | C_15_H_24_O | 2827752 | 97.1 | 0.12 | 1.23 |
| 77 | 28.2832 | (1R,3E,7E,11R)-1,5,5,8-Tetramethyl-12oxabicyclo[9.1.0]dodeca-3,7-diene | 19888-34-7 | C_15_H_24_O | 21578924 | 98.7 | 0.9 | 9.4 |
| 78 | 28.3881 | Selin-11-en-4-alpha-ol | 16641-47-7 | C_15_H_26_O | 976975 | 83.8 | 0.04 | 0.43 |
| 79 | 28.5699 | Selin-6-en-4.alpha.-ol | 118173-08-3 | C_15_H_26_O | 819323 | 71.5 | 0.03 | 0.36 |
| 80 | 28.7425 | Cedroxyde | 13786-79-3 | C_15_H_24_O | 2336084 | 85 | 0.1 | 1.02 |
| 81 | 29.3655 | Pyrimidine, 4-butyl-3,4-dihydro-5-methyl | 1000115-50-2 | C_9_H_16_N_2_ | 442264 | 72.5 | 0.02 | 0.19 |
| 82 | 29.4229 | Intermedeol | 6168-59-8 | C_15_H_26_O | 2416026 | 80.7 | 0.1 | 1.05 |
| 83 | 29.423 | 1-Naphthalenol,decahydro-1,4a-dimethyl-7-(1methylethylidene)-,[1R-(1.alpha.,4a.beta.,8a.alpha.)]- | 473-04-1 | C_15_H_26_O | 2640653 | 83.3 | 0.11 | 1.15 |
| 84 | 29.5175 | 6,9-Heptadecadiene | 81265-03-4 | C_17_H_32_ | 683607 | 85.1 | 0.03 | 0.3 |
| 85 | 29.6055 | 5-Heptadecene, 1-bromo | 56600-21-6 | C_17_H_33_Br | 4918141 | 86.6 | 0.21 | 2.14 |
| 86 | 29.7299 | 3-Heptadecene, (Z) | 1000141-67-3 | C_17_H_34_ | 130026752 | 97.9 | 5.43 | 56.66 |
| 87 | 29.8023 | 1,13-Tetradecadiene | 21964-49-8 | C_14_H_26_ | 3940647 | 91.7 | 0.16 | 1.72 |
| 88 | 29.9292 | 3-Heptadecene, (Z) | 1000141-67-3 | C_17_H_34_ | 198841248 | 97.8 | 8.3 | 86.65 |
| 89 | 30.0789 | 8-Heptadecene | 248097 | C_17_H_34_ | 4146748 | 96.3 | 0.17 | 1.81 |
| 90 | 30.0789 | 3-Heptadecene, (Z) | 1000141-67-3 | C_17_H_34_ | 4119186 | 96.8 | 0.17 | 1.8 |
| 91 | 30.2087 | Heptadecane | 629-78-7 | C_17_H_36_ | 172394124 | 99.5 | 7.2 | 75.13 |
| 92 | 31.7341 | Uvidin C, diacetate | 1000501-90-0 | C_19_H_30_O_5_ | 1063814 | 84.5 | 0.04 | 0.46 |
| 93 | 32.2926 | Decane, 3,8-dimethyl | 17312-55-9 | C_12_H_26_ | 390642 | 77.1 | 0.02 | 0.17 |
| 94 | 32.405 | cis-4-Decenal | 21662-09-9 | C_10_H_18_O | 240050 | 77.1 | 0.01 | 0.1 |
| 95 | 32.4059 | Pentanoic acid, 10-undecenyl ester | 1000159-93-4 | C_16_H_30_O_2_ | 240447 | 77.3 | 0.01 | 0.1 |
| 96 | 33.8216 | 11-Hexadecen-1-ol, (Z) | 56683-54-6 | C_16_H_32_O | 343030 | 88.1 | 0.01 | 0.15 |
| 97 | 33.8218 | 1,13-Tetradecadiene | 21964-49-8 | C_14_H_26_ | 340314 | 92.1 | 0.01 | 0.15 |
| 98 | 33.904 | Z-5-Nonadecene | 1000131-11-8 | C_19_H_38_ | 16094959 | 97.9 | 0.67 | 7.01 |
| 99 | 34.0125 | 1,15-Hexadecadiene | 21964-51-2 | C_16_H_30_ | 2453848 | 92.3 | 0.1 | 1.07 |
| 100 | 34.0997 | Z-5-Nonadecene | 1000131-11-8 | C_19_H_38_ | 29598082 | 97.8 | 1.24 | 12.9 |
| 101 | 34.2393 | 9-Nonadecene | 31035-07-1 | C_19_H_38_ | 712819 | 94.4 | 0.03 | 0.31 |
| 102 | 34.2395 | 1-Nonadecene | 18435-45-5 | C_19_H_38_ | 709242 | 95.7 | 0.03 | 0.31 |
| 103 | 34.3287 | Nonadecane | 629-92-5 | C_19_H_40_ | 12607296 | 99 | 0.53 | 5.49 |
| 104 | 35.5946 | Dibutyl phthalate | 84-74-2 | C_16_H_22_O_4_ | 406139 | 93.4 | 0.02 | 0.18 |
| 105 | 35.6965 | (E,E,E)-3,7,11,15-Tetramethylhexadeca-1,3,6,10,14-pentaene | 77898-97-6 | C_20_H_32_ | 262250 | 78.2 | 0.01 | 0.11 |
| 106 | 36.2811 | 5-Eicosene, (E) | 74685-30-6 | C_20_H_40_ | 292442 | 84.6 | 0.01 | 0.13 |
| 107 | 36.2835 | 7-Hexadecenal, (Z) | 56797-40-1 | C_16_H_30_O | 284773 | 88 | 0.01 | 0.12 |
| 108 | 36.4712 | 13-Octadecenal, (Z) | 58594-45-9 | C_18_H_34_O | 295800 | 91.7 | 0.01 | 0.13 |
| 109 | 36.6689 | Octadecanal | 638-66-4 | C_18_H_36_O | 690926 | 95.7 | 0.03 | 0.3 |
| 110 | 37.7415 | (2E,4E)-Methyl 5-(benzo[d][1,3]dioxol-5-yl)penta-2,4-dienoate | 6190-46-1 | C_13_H_12_O_4_ | 470951 | 81.2 | 0.02 | 0.21 |
| 111 | 38.1242 | Heneicosane | 629-94-7 | C_21_H_44_ | 601248 | 96.2 | 0.03 | 0.26 |

**Table S3.** Identification of major components of Asarum oil

| **No.** | **RT** | **Compound Name** | **CAS#** | **Formula** | **Area MI** | **Match Score** | **Area%-T** | **Area%-M** |
| --- | --- | --- | --- | --- | --- | --- | --- | --- |
| 1 | 5.2602 | Hexanal | 66-25-1 | C_6_H_12_O | 571209 | 97.9 | 0.04 | 0.2 |
| 2 | 6.8355 | Butanoic acid, 3-methyl-, ethyl ester | 108-64-5 | C_7_H_14_O_2_ | 460849 | 97.8 | 0.03 | 0.16 |
| 3 | 9.0786 | Bicyclo[3.1.0]hex-2-ene, 2-methyl-5-(1-methylethyl) | 353313 | C_10_H_16_ | 4717921 | 98.6 | 0.3 | 1.66 |
| 4 | 9.2769 | .alpha.-Pinene | 80-56-8 | C_10_H_16_ | 53606563 | 99.1 | 3.36 | 18.92 |
| 5 | 9.7379 | Camphene | 79-92-5 | C_10_H_16_ | 14019438 | 99.2 | 0.88 | 4.95 |
| 6 | 10.5841 | Bicyclo[3.1.0]hexane, 4-methylene-1-(1-methylethyl) | 3387-41-5 | C_10_H_16_ | 5142709 | 99 | 0.32 | 1.81 |
| 7 | 10.6589 | Bicyclo[3.1.1]heptane, 6,6-dimethyl-2-methylene-, (1S) | 18172-67-3 | C_10_H_16_ | 51136901 | 98.8 | 3.21 | 18.05 |
| 8 | 10.9879 | Hexanoic acid | 142-62-1 | C_6_H_12_O_2_ | 288431 | 90.8 | 0.02 | 0.1 |
| 9 | 11.1886 | .beta.-Myrcene | 123-35-3 | C_10_H_16_ | 11780053 | 98.7 | 0.74 | 4.16 |
| 10 | 11.5761 | .alpha.-Phellandrene | 99-83-2 | C_10_H_16_ | 31744679 | 97.2 | 1.99 | 11.2 |
| 11 | 11.6996 | Isobutyl isovalerate | 589-59-3 | C_9_H_18_O_2_ | 472069 | 84.6 | 0.03 | 0.17 |
| 12 | 11.7683 | 3-Carene | 13466-78-9 | C_10_H_16_ | 97489367 | 98.5 | 6.12 | 34.4 |
| 13 | 11.7701 | 3-Hydroxy-4-methylbenzaldehyde | 57295-30-4 | C_8_H_8_O_2_ | 7407674 | 71 | 0.46 | 2.61 |
| 14 | 11.9734 | 1,3-Cyclohexadiene, 1-methyl-4-(1-methylethyl) | 99-86-5 | C_10_H_16_ | 1500144 | 97.4 | 0.09 | 0.53 |
| 15 | 12.1557 | o-Cymene | 527-84-4 | C_10_H_14_ | 648239 | 97.7 | 0.04 | 0.23 |
| 16 | 12.233 | p-Cymene | 99-87-6 | C_10_H_14_ | 17310360 | 98.3 | 1.09 | 6.11 |
| 17 | 12.3625 | D-Limonene | 5989-27-5 | C_10_H_16_ | 22595221 | 96.8 | 1.42 | 7.97 |
| 18 | 12.4307 | Eucalyptol | 470-82-6 | C_10_H_18_O | 17253494 | 99.7 | 1.08 | 6.09 |
| 19 | 12.6953 | trans-.beta.-Ocimene | 3779-61-1 | C_10_H_16_ | 3246315 | 99.4 | 0.2 | 1.15 |
| 20 | 13.0169 | .beta.-Ocimene | 13877-91-3 | C_10_H_16_ | 1740487 | 99 | 0.11 | 0.61 |
| 21 | 13.2588 | .alpha.-Phellandrene | 99-83-2 | C_10_H_16_ | 325092 | 94.5 | 0.02 | 0.11 |
| 22 | 13.3312 | .gamma.-Terpinene | 99-85-4 | C_10_H_16_ | 3805381 | 99.4 | 0.24 | 1.34 |
| 23 | 14.1887 | Cyclohexene, 3-methyl-6-(1-methylethylidene) | 586-63-0 | C_10_H_16_ | 2504436 | 97.5 | 0.16 | 0.88 |
| 24 | 14.2557 | Cyclohexene, 1-methyl-4-(1-methylethylidene) | 586-62-9 | C_10_H_16_ | 12939672 | 98.7 | 0.81 | 4.57 |
| 25 | 14.2838 | Benzene, 4-ethenyl-1,2-dimethyl | 27831-13-6 | C_10_H_12_ | 1176148 | 89.3 | 0.07 | 0.42 |
| 26 | 14.2838 | Benzaldehyde, 4-(1-phenyl-2-propenyloxy) | 1000277-56-1 | C_16_H_14_O_2_ | 580112 | 71.5 | 0.04 | 0.2 |
| 27 | 14.6369 | Ethyllinalool | 1000430-72-3 | C_11_H_20_O | 442395 | 83.2 | 0.03 | 0.16 |
| 28 | 14.6491 | Linalool | 78-70-6 | C_10_H_18_O | 691677 | 83.6 | 0.04 | 0.24 |
| 29 | 15.1362 | (E)-4,8-Dimethylnona-1,3,7-triene | 19945-61-0 | C_11_H_18_ | 607596 | 88 | 0.04 | 0.21 |
| 30 | 15.3065 | 2-Cyclohexen-1-ol, 1-methyl-4-(1-methylethyl)-, trans- | 29803-81-4 | C_10_H_18_O | 297645 | 73.7 | 0.02 | 0.11 |
| 31 | 15.6991 | cis-p-Mentha-2,8-dien-1-ol | 3886-78-0 | C_10_H_16_O | 313560 | 85.1 | 0.02 | 0.11 |
| 32 | 15.9648 | (+)-2-Bornanone | 464-49-3 | C_10_H_16_O | 2787098 | 99.5 | 0.17 | 0.98 |
| 33 | 16.2224 | 2,4-Cycloheptadien-1-one, 2,6,6-trimethyl | 503-93-5 | C_10_H_14_O | 138118344 | 99.3 | 8.66 | 48.74 |
| 34 | 16.4907 | p-Mentha-1,5-dien-8-ol | 1686-20-0 | C_10_H_16_O | 307902 | 84.2 | 0.02 | 0.11 |
| 35 | 16.5203 | Pinocarvone | 30460-92-5 | C_10_H_14_O | 378959 | 76.9 | 0.02 | 0.13 |
| 36 | 16.678 | p-Mentha-1,5-dien-8-ol | 1686-20-0 | C_10_H_16_O | 356993 | 85.5 | 0.02 | 0.13 |
| 37 | 16.6912 | Cyclohexene, 3-acetoxy-4-(1-hydroxy-1-methylethyl)1-methyl | 1000196-01-7 | C_12_H_20_O_3_ | 863284 | 79.9 | 0.05 | 0.3 |
| 38 | 16.7994 | Carvenone | 499-74-1 | C_10_H_16_O | 283818 | 78.4 | 0.02 | 0.1 |
| 39 | 16.9664 | 3-Cyclohexen-1-ol, 4-methyl-1-(1-methylethyl)-, (R) | 20126-76-5 | C_10_H_18_O | 7249857 | 98.4 | 0.45 | 2.56 |
| 40 | 17.2098 | Benzylephedrine | 139253-56-8 | C_17_H_21_NO | 633654 | 77.1 | 0.04 | 0.22 |
| 41 | 17.2111 | m-Cymen-8-ol | 5208-37-7 | C_10_H_14_O | 2067216 | 88.6 | 0.13 | 0.73 |
| 42 | 17.2114 | m-Cymen-8-ol | 5208-37-7 | C_10_H_14_O | 1952601 | 90.1 | 0.12 | 0.69 |
| 43 | 17.388 | .alpha.-Terpineol | 98-55-5 | C_10_H_18_O | 5973559 | 98.5 | 0.37 | 2.11 |
| 44 | 17.5844 | Estragole | 140-67-0 | C_10_H_12_O | 20820901 | 99.6 | 1.31 | 7.35 |
| 45 | 17.8837 | 2,4-Cycloheptadien-1-one, 2,6,6-trimethyl | 503-93-5 | C_10_H_14_O | 924665 | 95.7 | 0.06 | 0.33 |
| 46 | 18.2297 | 1-Cyclohexene-1-carboxaldehyde, 2,6,6-trimethyl | 432-25-7 | C_10_H_16_O | 426862 | 88.9 | 0.03 | 0.15 |
| 47 | 18.6265 | Benzene, 2-methoxy-4-methyl-1-(1-methylethyl) | 1076-56-8 | C_11_H_16_O | 2794540 | 98.1 | 0.18 | 0.99 |
| 48 | 18.6268 | Benzene, 1-methoxy-4-methyl-2-(1-methylethyl) | 31574-44-4 | C_11_H_16_O | 2808361 | 97.3 | 0.18 | 0.99 |
| 49 | 18.895 | Benzene, 2-methoxy-1-methyl-4-(1-methylethyl) | 6379-73-3 | C_11_H_16_O | 452485 | 85.2 | 0.03 | 0.16 |
| 50 | 19.1168 | 4,7,7-Trimethylbicyclo[4.1.0]hept-3-en-2-one | 81800-50-2 | C_10_H_14_O | 2383533 | 96.9 | 0.15 | 0.84 |
| 51 | 19.1888 | 2-Cyclohexen-1-one, 3-methyl-6-(1-methylethyl) | 89-81-6 | C_10_H_16_O | 559754 | 81.7 | 0.04 | 0.2 |
| 52 | 19.5738 | 2-Methoxy-6-methyl-cyclohexa-2,5-dienecarboxamide | 1000189-91-0 | C_9_H_13_NO_2_ | 189053975 | 81.7 | 11.86 | 66.72 |
| 53 | 19.7144 | 2-Cyclohexen-1-one, 3-methyl-6-(1-methylethenyl)-, (S) | 16750-82-6 | C_10_H_14_O | 712658 | 87.7 | 0.04 | 0.25 |
| 54 | 20.2195 | Safrole | 94-59-7 | C_10_H_10_O_2_ | 283366101 | 77.5 | 17.78 | 100 |
| 55 | 20.2891 | Pentandioic acid, (p-t-butylphenyl) ester | 212762-88-4 | C_15_H_20_O_4_ | 324618 | 78.7 | 0.02 | 0.11 |
| 56 | 20.44 | Decanoic acid, 2-phenylethyl ester | 61810-55-7 | C_18_H_28_O_2_ | 317337 | 70.8 | 0.02 | 0.11 |
| 57 | 20.5708 | Pentandioic acid, (p-t-butylphenyl) ester | 212762-88-4 | C_15_H_20_O_4_ | 290145 | 78.4 | 0.02 | 0.1 |
| 58 | 20.8355 | 2-Methoxy-4-vinylphenol | 7786-61-0 | C_9_H_10_O_2_ | 1397569 | 97 | 0.09 | 0.49 |
| 59 | 20.8407 | Benzene, nitroso | 586-96-9 | C_6_H_5_NO | 397770 | 76.3 | 0.02 | 0.14 |
| 60 | 21.061 | 3,7,7-Trimethylbicyclo[4.1.0]hept-3-ene-2,5-dione | 6617-34-1 | C_10_H_12_O_2_ | 1360001 | 97.7 | 0.09 | 0.48 |
| 61 | 21.7846 | .alpha.-Terpinyl acetate | 80-26-2 | C_12_H_20_O_2_ | 579467 | 96.6 | 0.04 | 0.2 |
| 62 | 22.0101 | Eugenol | 97-53-0 | C_10_H_12_O_2_ | 1164142 | 96.3 | 0.07 | 0.41 |
| 63 | 22.4571 | 5-Azulenemethanol,1,2,3,4,5,6,7,8-octahydro.alpha.,.alpha.,3,8-tetramethyl-, acetate, [3S(3.alpha.,5.alpha.,8.alpha.)] | 134-28-1 | C_17_H_28_O_2_ | 321339 | 71.1 | 0.02 | 0.11 |
| 64 | 22.5187 | Safrole | 94-59-7 | C_10_H_10_O_2_ | 302312 | 82.6 | 0.02 | 0.11 |
| 65 | 22.6014 | .gamma.-Muurolene | 30021-74-0 | C_15_H_24_ | 1177582 | 87.4 | 0.07 | 0.42 |
| 66 | 22.726 | .beta.-GURJUNENE | 1000425-17-9 | C_15_H_24_ | 323922 | 77.3 | 0.02 | 0.11 |
| 67 | 22.9348 | Cyclohexane, 1-ethenyl-1-methyl-2,4-bis(1methylethenyl)-, [1S-(1.alpha.,2.beta.,4.beta.)] | 515-13-9 | C_15_H_24_ | 653977 | 94.2 | 0.04 | 0.23 |
| 68 | 23.3352 | Benzene, 1,2,3-trimethoxy-5-methyl | 6443-69-2 | C_10_H_14_O_3_ | 135280351 | 93 | 8.49 | 47.74 |
| 69 | 23.4648 | Benzene, 1,2,3-trimethoxy-5-methyl | 6443-69-2 | C_10_H_14_O_3_ | 109935917 | 95.7 | 6.9 | 38.8 |
| 70 | 23.5494 | (1R,2S,6S,7S,8S)-8-Isopropyl-1-methyl-3methylenetricyclo[4.4.0.02,7]decane-rel | 18252-44-3 | C_15_H_24_ | 2373918 | 87.4 | 0.15 | 0.84 |
| 71 | 23.657 | (-)-Aristolene | 6831-16-9 | C_15_H_24_ | 2133049 | 95.5 | 0.13 | 0.75 |
| 72 | 23.8283 | 1-Propanone, 2-chloro-1-(2,4-dimethylphenyl)-2methyl | 54965-53-6 | C_12_H_15_ClO | 987050 | 77 | 0.06 | 0.35 |
| 73 | 23.993 | 1H-Cyclopropa[a]naphthalene,1a,2,3,5,6,7,7a,7boctahydro-1,1,7,7a-tetramethyl-, [1aR(1a.alpha.,7.alpha.,7a.alpha.,7b.alpha.)] | 17334-55-3 | C_15_H_24_ | 5161852 | 94.9 | 0.32 | 1.82 |
| 74 | 24.0473 | trans-.alpha.-Bergamotene | 13474-59-4 | C_15_H_24_ | 1891950 | 77.9 | 0.12 | 0.67 |
| 75 | 24.1333 | .alpha.-Guaiene | 654486 | C_15_H_24_ | 1288329 | 94 | 0.08 | 0.45 |
| 76 | 24.2596 | (1R,3aS,8aS)-7-Isopropyl-1,4-dimethyl-1,2,3,3a,6,8ahexahydroazulene | 36577-33-0 | C_15_H_24_ | 5946399 | 89.6 | 0.37 | 2.1 |
| 77 | 24.2712 | Bromonitromethane | 563-70-2 | CH_2_BrNO_2_ | 1727717 | 76.9 | 0.11 | 0.61 |
| 78 | 24.3187 | Ethanone, 1,1',1''-(1,3,5-benzenetriyl)tris | 779-90-8 | C_12_H_12_O_3_ | 660699 | 74.2 | 0.04 | 0.23 |
| 79 | 24.3191 | 3-Methoxy-2,4,5-trifluorobenzoic acid, 2-formyl-4,6dichlorophenyl ester | 1000331-59-5 | C_15_H_7_C_l2_F_3_O_4_ | 322235 | 78.1 | 0.02 | 0.11 |
| 80 | 24.3948 | 1-Propanone, 1-(4-methoxyphenyl) | 121-97-1 | C_10_H_12_O_2_ | 741796 | 78.5 | 0.05 | 0.26 |
| 81 | 24.4047 | 2H-2,4a-Ethanonaphthalene, 1,3,4,5,6,7-hexahydro2,5,5-trimethyl | 32391-44-9 | C_15_H_24_ | 1058151 | 70.3 | 0.07 | 0.37 |
| 82 | 24.4047 | p-Hexylacetophenone | 37592-72-6 | C_14_H_20_O | 1058732 | 70.4 | 0.07 | 0.37 |
| 83 | 24.5299 | Caryophyllene | 87-44-5 | C_15_H_24_ | 1454239 | 85.7 | 0.09 | 0.51 |
| 84 | 24.666 | 1,3-Benzodioxole, 4-methoxy-6-(2-propenyl) | 607-91-0 | C_11_H_12_O_3_ | 29924205 | 98.1 | 1.88 | 10.56 |
| 85 | 24.7161 | 2,3-Dimethoxy-5-methylhydroquinone, O,O'-dimethyl | 35896-58-3 | C_11_H_16_O_4_ | 558465 | 83.3 | 0.04 | 0.2 |
| 86 | 24.9633 | (1R,2S,6S,7S,8S)-8-Isopropyl-1-methyl-3methylenetricyclo[4.4.0.02,7]decane-rel | 18252-44-3 | C_15_H_24_ | 4260687 | 89.9 | 0.27 | 1.5 |
| 87 | 25.2533 | 1-Pentadecene | 13360-61-7 | C_15_H_30_ | 424004 | 92.6 | 0.03 | 0.15 |
| 88 | 25.3846 | (1S,4S,4aS)-1-Isopropyl-4,7-dimethyl-1,2,3,4,4a,5hexahydronaphthalene | 267665-20-3 | C_15_H_24_ | 2542676 | 89 | 0.16 | 0.9 |
| 89 | 25.4144 | (3R,5aR,9S,9aS)-2,2,5a,9-Tetramethyloctahydro-2H3,9a-methanobenzo[b]oxepine | 765307-45-7 | C_15_H_26_O | 3625685 | 91.2 | 0.23 | 1.28 |
| 90 | 25.5644 | Pentadecane | 629-62-9 | C_15_H_32_ | 8900820 | 81 | 0.56 | 3.14 |
| 91 | 25.5645 | Hexadecane | 544-76-3 | C_16_H_34_ | 8972286 | 79.9 | 0.56 | 3.17 |
| 92 | 25.5692 | Asaricin | 18607-93-7 | C_11_H_12_O_3_ | 101352892 | 95.3 | 6.36 | 35.77 |
| 93 | 25.6845 | (1R,3aS,4aS,8aS)-1,4,4,6-Tetramethyl1,2,3,3a,4,4a,7,8octahydrocyclopenta[1,4]cyclobuta[1,2]benzene | 94535-52-1 | C_15_H_24_ | 1674224 | 91.8 | 0.11 | 0.59 |
| 94 | 25.8184 | Azulene, 1,2,3,5,6,7,8,8a-octahydro-1,4-dimethyl-7(1-methylethenyl)-, [1S-(1.alpha.,7.alpha.,8a.beta.)] | 3691-11-0 | C_15_H_24_ | 4082508 | 96.4 | 0.26 | 1.44 |
| 95 | 25.9416 | Butylated Hydroxytoluene | 128-37-0 | C_15_H_24_O | 820895 | 94.1 | 0.05 | 0.29 |
| 96 | 26.1567 | 1,3-Benzodioxole, 4-methoxy-6-(2-propenyl) | 607-91-0 | C_11_H_12_O_3_ | 6691818 | 98.1 | 0.42 | 2.36 |
| 97 | 26.2182 | 1-Isopropyl-4,7-dimethyl-1,2,3,5,6,8ahexahydronaphthalene | 16729-01-4 | C_15_H_24_ | 1339141 | 87.2 | 0.08 | 0.47 |
| 98 | 26.3129 | 2,3-Dimethoxy-5-methylhydroquinone, O,O'-dimethyl | 35896-58-3 | C_11_H_16_O_4_ | 685407 | 82.5 | 0.04 | 0.24 |
| 99 | 26.4135 | 3,4-Methylenedioxypropiophenone | 28281-49-4 | C_10_H_10_O_3_ | 12207378 | 98.3 | 0.77 | 4.31 |
| 100 | 26.9668 | Benzene, 1,2,3-trimethoxy-5-(2-propenyl) | 487-11-6 | C_12_H_16_O_3_ | 59861179 | 99 | 3.76 | 21.13 |
| 101 | 27.1709 | 1,6,10-Dodecatrien-3-ol, 3,7,11-trimethyl-, (E) | 40716-66-3 | C_15_H_26_O | 1812402 | 89 | 0.11 | 0.64 |
| 102 | 27.3729 | Benzene, 1,2,3-trimethoxy-5-(1-propenyl)-, (E) | 5273-85-8 | C_12_H_16_O_3_ | 2471556 | 97.8 | 0.16 | 0.87 |
| 103 | 27.6648 | (-)-Spathulenol | 77171-55-2 | C_15_H_24_O | 606800 | 72.9 | 0.04 | 0.21 |
| 104 | 27.8478 | 2-Hydroxy-4,5-methylenedioxypropiophenone | 18607-90-4 | C_10_H_10_O_4_ | 998839 | 92.9 | 0.06 | 0.35 |
| 105 | 28.8045 | 2-Hydroxy-4,5-methylenedioxypropiophenone | 18607-90-4 | C_10_H_10_O_4_ | 17198261 | 96.9 | 1.08 | 6.07 |
| 106 | 29.4274 | Patchouli alcohol | 5986-55-0 | C_15_H_26_O | 1567851 | 93.6 | 0.1 | 0.55 |
| 107 | 29.6316 | Xanthoxylin | 90-24-4 | C_10_H_12_O_4_ | 532020 | 77 | 0.03 | 0.19 |
| 108 | 29.7047 | E-14-Hexadecenal | 330207-53-9 | C_16_H_30_O | 487295 | 84.1 | 0.03 | 0.17 |
| 109 | 29.7057 | 2-Methyl-E-7-hexadecene | 64183-52-4 | C_17_H_34_ | 301987 | 85.3 | 0.02 | 0.11 |
| 110 | 29.8817 | 8-Heptadecene | 248097 | C_17_H_34_ | 370974 | 81.8 | 0.02 | 0.13 |
| 111 | 29.9246 | Bisabolol acetate,alpha | 25428-44-8 | C_17_H_28_O_2_ | 608873 | 72.4 | 0.04 | 0.21 |
| 112 | 30.1669 | Heptadecane | 629-78-7 | C_17_H_36_ | 421617 | 95.7 | 0.03 | 0.15 |
| 113 | 31.0439 | 2',4'-Dimethoxy-3'-methylpropiophenone | 77942-13-3 | C_12_H_16_O_3_ | 4689605 | 82.7 | 0.29 | 1.65 |
| 114 | 31.6281 | 2,4a-Methanonaphthalen-7(4aH)-one, 1,2,3,4,5,6hexahydro-1,1,5,5-tetramethyl-, (2s-cis) | 26839-52-1 | C_15_H_22_O | 425372 | 86 | 0.03 | 0.15 |
| 115 | 31.629 | 2H-Cyclopropa[a]naphthalen-2-one, 1,1a,4,5,6,7,7a,7b-octahydro-1,1,7,7a-tetramethyl-, (1a.alpha.,7.alpha.,7a.alpha.,7b.alpha.) | 6831-17-0 | C_15_H_22_O | 450041 | 81.1 | 0.03 | 0.16 |
| 116 | 35.5937 | Dibutyl phthalate | 84-74-2 | C_16_H_22_O_4_ | 432331 | 97.2 | 0.03 | 0.15 |

**Table S4**. Identification of major components of Galangal oil

| **No.** | **RT** | **Compound Name** | **CAS#** | **Formula** | **Area MI** | **Match Score** | **Area%-T** | **Area%-M** |
| --- | --- | --- | --- | --- | --- | --- | --- | --- |
| 1 | 4.6904 | Methyl isovalerate | 556-24-1 | C_6_H_12_O_2_ | 1013542 | 98.4 | 0.05 | 0.15 |
| 2 | 8.7325 | Propanoic acid, 2-methyl-, 2-methylpropyl ester | 97-85-8 | C_8_H_16_O_2_ | 9780786 | 99.7 | 0.45 | 1.49 |
| 3 | 8.8753 | Tricyclo[2.2.1.0(2,6)]heptane, 1,7,7-trimethyl | 508-32-7 | C_10_H_16_ | 2638364 | 98.5 | 0.12 | 0.4 |
| 4 | 9.0803 | Bicyclo[3.1.0]hex-2-ene, 2-methyl-5-(1-methylethyl) | 353313 | C_10_H_16_ | 2691125 | 98.7 | 0.12 | 0.41 |
| 5 | 9.283 | α-Pinene | 80-56-8 | C_10_H_16_ | 102616685 | 99 | 4.69 | 15.64 |
| 6 | 9.7493 | Camphene | 79-92-5 | C_10_H_16_ | 108857441 | 99.2 | 4.98 | 16.59 |
| 7 | 10.0681 | Butanoic acid, 2-methylpropyl ester | 539-90-2 | C_8_H_16_O_2_ | 824387 | 97.8 | 0.04 | 0.13 |
| 8 | 10.6691 | Bicyclo[3.1.1]heptane, 6,6-dimethyl-2-methylene-, (1S) | 18172-67-3 | C_10_H_16_ | 119137245 | 98.6 | 5.45 | 18.16 |
| 9 | 11.0633 | 5-Hepten-2-one, 6-methyl | 110-93-0 | C_8_H_14_O | 15070895 | 98.8 | 0.69 | 2.3 |
| 10 | 11.1896 | β-Myrcene | 123-35-3 | C_10_H_16_ | 3837320 | 97.6 | 0.18 | 0.58 |
| 11 | 11.5764 | α-Phellandrene | 99-83-2 | C_10_H_16_ | 1698837 | 91.3 | 0.08 | 0.26 |
| 12 | 11.585 | Butanoic acid, 2-methyl-, 2-methylpropyl ester | 2445-67-2 | C_9_H_18_O_2_ | 6445698 | 98.1 | 0.29 | 0.98 |
| 13 | 11.7003 | Isobutyl isovalerate | 589-59-3 | C_9_H_18_O_2_ | 2364082 | 98.3 | 0.11 | 0.36 |
| 14 | 11.9119 | Propanoic acid, 2-methyl-, 3-methylbutyl ester | 54791 | C_9_H_18_O_2_ | 3222604 | 98 | 0.15 | 0.49 |
| 15 | 11.981 | 1,3-Cyclohexadiene, 1-methyl-4-(1-methylethyl) | 99-86-5 | C_10_H_16_ | 5104693 | 95.3 | 0.23 | 0.78 |
| 16 | 12.0308 | Propanoic acid, 2-methyl-, 2-methylbutyl ester | 2445-69-4 | C_9_H_18_O_2_ | 3344598 | 97.3 | 0.15 | 0.51 |
| 17 | 12.2652 | o-Cymene | 527-84-4 | C_10_H_14_ | 17098852 | 97.3 | 0.78 | 2.61 |
| 18 | 12.2652 | p-Cymene | 99-87-6 | C_10_H_14_ | 17040811 | 97.4 | 0.78 | 2.6 |
| 19 | 12.4584 | D-Limonene | 5989-27-5 | C_10_H_16_ | 123797935 | 83.3 | 5.66 | 18.87 |
| 20 | 12.4636 | Cyanogen chloride | 506-77-4 | CClN | 1331782 | 72.4 | 0.06 | 0.2 |
| 21 | 12.5007 | 2-Butanone | 78-93-3 | C_4_H_8_O | 57426243 | 82.5 | 2.63 | 8.75 |
| 22 | 12.5283 | Eucalyptol | 470-82-6 | C_10_H_18_O | 656186341 | 93.9 | 30.01 | 100 |
| 23 | 13.1941 | 5-Heptenal, 2,6-dimethyl | 106-72-9 | C_9_H_16_O | 613286 | 86.9 | 0.03 | 0.09 |
| 24 | 13.2781 | Butanoic acid, 3-methylbutyl ester | 106-27-4 | C_9_H_18_O_2_ | 693081 | 92.1 | 0.03 | 0.11 |
| 25 | 13.3356 | .gamma.-Terpinene | 99-85-4 | C_10_H_16_ | 9707789 | 99.1 | 0.44 | 1.48 |
| 26 | 13.3564 | Butanoic acid, 2-methylbutyl ester | 51115-64-1 | C_9_H_18_O_2_ | 879734 | 74.7 | 0.04 | 0.13 |
| 27 | 13.7491 | (2S)-2-(Acetyloxy)-3-methylbutanoic acid | 18667-97-5 | C_7_H_12_O_4_ | 1376904 | 77 | 0.06 | 0.21 |
| 28 | 14.2572 | Cyclohexene, 1-methyl-4-(1-methylethylidene) | 586-62-9 | C_10_H_16_ | 7745021 | 97.6 | 0.35 | 1.18 |
| 29 | 14.2867 | Benzene, 4-ethenyl-1,2-dimethyl | 27831-13-6 | C_10_H_12_ | 872383 | 85.4 | 0.04 | 0.13 |
| 30 | 14.6375 | 1,5-Dimethyl-1-vinyl-4-hexenyl butyrate | 78-36-4 | C_14_H_24_O_2_ | 6117547 | 81.9 | 0.28 | 0.93 |
| 31 | 14.6471 | Linalool | 78-70-6 | C_10_H_18_O | 10196760 | 89.5 | 0.47 | 1.55 |
| 32 | 14.6475 | Butanoic acid, 2-methyl-, 3-methylbutyl ester | 27625-35-0 | C_10_H_20_O_2_ | 6786994 | 89.6 | 0.31 | 1.03 |
| 33 | 14.764 | Butanoic acid, 2-methyl-, 2-methylbutyl ester | 2445-78-5 | C_10_H_20_O_2_ | 3133913 | 98.5 | 0.14 | 0.48 |
| 34 | 14.8076 | Butanoic acid, 3-methyl-, 3-methylbutyl ester | 659-70-1 | C_10_H_20_O_2_ | 1145722 | 83.4 | 0.05 | 0.17 |
| 35 | 14.8947 | Butanoic acid, 3-methyl-, 2-methylbutyl ester | 2445-77-4 | C_10_H_20_O_2_ | 2493388 | 98.8 | 0.11 | 0.38 |
| 36 | 15.3293 | p-Menth-2-en-7-ol, cis | 19898-86-3 | C_10_H_18_O | 756991 | 83.3 | 0.03 | 0.12 |
| 37 | 15.9661 | (+)-2-Bornanone | 464-49-3 | C_10_H_16_O | 47630835 | 99.8 | 2.18 | 7.26 |
| 38 | 16.0934 | Bicyclo[2.2.1]heptan-2-ol, 2,3,3-trimethyl | 465-31-6 | C_10_H_18_O | 3226143 | 94.9 | 0.15 | 0.49 |
| 39 | 16.4489 | 2-Hydroxyisocaproic acid, acetate | 1000374-89-3 | C_8_H_14_O_4_ | 4835398 | 79.6 | 0.22 | 0.74 |
| 40 | 16.529 | Bicyclo[2.2.1]heptane, 2-chloro-1,7,7-trimethyl-, (1Rendo) | 30462-53-4 | C_10_H_17_Cl | 2126077 | 90.6 | 0.1 | 0.32 |
| 41 | 16.68 | Cyclohexanemethanol, α,α-dimethyl-4-methylene | 7299-42-5 | C_10_H_18_O | 7330380 | 96.7 | 0.34 | 1.12 |
| 42 | 16.9751 | 3-Cyclohexen-1-ol, 4-methyl-1-(1-methylethyl)-, (R) | 20126-76-5 | C_10_H_18_O | 42238503 | 99.2 | 1.93 | 6.44 |
| 43 | 17.2125 | Dill ether | 74410-10-9 | C_10_H_16_O | 776702 | 78.6 | 0.04 | 0.12 |
| 44 | 17.4127 | α-Terpineol | 98-55-5 | C_10_H_18_O | 167195625 | 98.2 | 7.65 | 25.48 |
| 45 | 18.2156 | Fenchyl acetate | 13851-11-1 | C_12_H_20_O_2_ | 15049957 | 99.2 | 0.69 | 2.29 |
| 46 | 18.7254 | 2-Tetrahydrofurfuryl isothiocyanate | 36810-87-4 | C_6_H_9_NOS | 659509 | 74.5 | 0.03 | 0.1 |
| 47 | 18.7254 | Butanal diisopentyl acetal | 1000431-60-7 | C_14_H_30_O_2_ | 554660 | 74.1 | 0.03 | 0.08 |
| 48 | 18.8765 | 2-Butanone, 4-phenyl | 2550-26-7 | C_10_H_12_O | 13749369 | 99.1 | 0.63 | 2.1 |
| 49 | 19.2506 | Acetic acid, 2-phenylethyl ester | 103-45-7 | C_10_H_12_O_2_ | 758744 | 96.9 | 0.03 | 0.12 |
| 50 | 19.8502 | 2,4-Dimethylpentan-3-yl 2-methylbutanoate | 1000372-73-2 | C_12_H_24_O_2_ | 2078120 | 76.9 | 0.1 | 0.32 |
| 51 | 20.0741 | Bicyclo[2.2.1]heptan-2-ol, 1,7,7-trimethyl-, acetate, (1S-endo) | 5655-61-8 | C_12_H_20_O_2_ | 4996305 | 97.5 | 0.23 | 0.76 |
| 52 | 21.2113 | Benzoic acid, 2-methylpropyl ester | 120-50-3 | C_11_H_14_O_2_ | 6073008 | 98.8 | 0.28 | 0.93 |
| 53 | 21.7835 | α-Terpinyl acetate | 80-26-2 | C_12_H_20_O_2_ | 625035 | 88.2 | 0.03 | 0.1 |
| 54 | 22.1517 | Valeric anhydride | 2082-59-9 | C_10_H_18_O_3_ | 938083 | 86.1 | 0.04 | 0.14 |
| 55 | 22.3049 | 1,2,4-Metheno-1H-indene, octahydro-1,7a-dimethyl-5(1-methylethyl)-, [1S(1.alpha.,2.alpha.,3a.beta.,4.alpha.,5.alpha.,7a.beta.,8 S*)] | 22469-52-9 | C_15_H_24_ | 1963708 | 98 | 0.09 | 0.3 |
| 56 | 22.401 | Ylangene | 14912-44-8 | C_15_H_24_ | 8270955 | 98.7 | 0.38 | 1.26 |
| 57 | 22.5194 | Copaene | 3856-25-5 | C_15_H_24_ | 3150312 | 98.6 | 0.14 | 0.48 |
| 58 | 22.9347 | Cyclohexane, 1-ethenyl-1-methyl-2,4-bis(1methylethenyl)-, [1S-(1.alpha.,2.beta.,4.beta.)] | 515-13-9 | C_15_H_24_ | 4434255 | 87.8 | 0.2 | 0.68 |
| 59 | 22.976 | Benzene, 4-ethenyl-1,2-dimethyl | 27831-13-6 | C_10_H_12_ | 1294722 | 70.8 | 0.06 | 0.2 |
| 60 | 22.9813 | Hydroxylamine, O-(phenylmethyl) | 622-33-3 | C_7_H_9_NO | 2369651 | 71.6 | 0.11 | 0.36 |
| 61 | 22.9833 | β-Phenylethyl butyrate | 103-52-6 | C_12_H_16_O_2_ | 9808538 | 96 | 0.45 | 1.49 |
| 62 | 23.5187 | cis-α-Bergamotene | 18252-46-5 | C_15_H_24_ | 1421676 | 97.4 | 0.07 | 0.22 |
| 63 | 23.6672 | Caryophyllene | 87-44-5 | C_15_H_24_ | 53075594 | 97.9 | 2.43 | 8.09 |
| 64 | 23.9024 | (1R,2S,6S,7S,8S)-8-Isopropyl-1-methyl-3methylenetricyclo[4.4.0.02,7]decane-rel | 18252-44-3 | C_15_H_24_ | 816381 | 96.3 | 0.04 | 0.12 |
| 65 | 24.0435 | cis-α-Bergamotene | 18252-46-5 | C_15_H_24_ | 47523926 | 98.2 | 2.17 | 7.24 |
| 66 | 24.1283 | α-Guaiene | 654486 | C_15_H_24_ | 9415628 | 97.9 | 0.43 | 1.43 |
| 67 | 24.1451 | Propanoic acid, 2-methyl-, 2-phenylethyl ester | 103-48-0 | C_12_H_16_O_2_ | 1244161 | 74.9 | 0.06 | 0.19 |
| 68 | 24.2481 | (1R,3aS,8aS)-7-Isopropyl-1,4-dimethyl-1,2,3,3a,6,8ahexahydroazulene | 36577-33-0 | C_15_H_24_ | 1860886 | 96.6 | 0.09 | 0.28 |
| 69 | 24.3983 | Isoledene | 95910-36-4 | C_15_H_24_ | 11843951 | 97 | 0.54 | 1.8 |
| 70 | 24.5295 | Humulene | 6753-98-6 | C_15_H_24_ | 15190629 | 99.3 | 0.69 | 2.31 |
| 71 | 24.6624 | Bicyclo[2.2.1]heptane, 2-methyl-3-methylene-2-(4methyl-3-pentenyl)-, (1S-exo) | 511-59-1 | C_15_H_24_ | 1869245 | 86.7 | 0.09 | 0.28 |
| 72 | 24.7059 | Cadina-1(6),4-diene, trans | 20085-11-4 | C_15_H_24_ | 868580 | 82.9 | 0.04 | 0.13 |
| 73 | 24.7609 | (+)-epi-Bicyclosesquiphellandrene | 54274-73-6 | C_15_H_24_ | 1115375 | 91.1 | 0.05 | 0.17 |
| 74 | 24.7612 | Bicyclosesquiphellandrene | 54324-03-7 | C_15_H_24_ | 1086523 | 92.1 | 0.05 | 0.17 |
| 75 | 25.0792 | .gamma.-Muurolene | 30021-74-0 | C_15_H_24_ | 10316603 | 96.7 | 0.47 | 1.57 |
| 76 | 25.0821 | Naphthalene, 1,2,3,4,4a,5,6,8a-octahydro-7-methyl-4methylene-1-(1-methylethyl)-, (1.alpha.,4a.beta.,8a.alpha.) | 39029-41-9 | C_15_H_24_ | 8003550 | 90 | 0.37 | 1.22 |
| 77 | 25.1756 | Naphthalene, 1,2,4a,5,6,8a-hexahydro-4,7-dimethyl-1(1-methylethyl) | 483-75-0 | C_15_H_24_ | 3174493 | 89.2 | 0.15 | 0.48 |
| 78 | 25.2075 | Benzene, 1-(1,5-dimethyl-4-hexenyl)-4-methyl | 644-30-4 | C_15_H_22_ | 1343950 | 71.2 | 0.06 | 0.2 |
| 79 | 25.311 | Pentanoic acid, 2-phenylethyl ester | 7460-74-4 | C_13_H_18_O_2_ | 6653474 | 88.2 | 0.3 | 1.01 |
| 80 | 25.3478 | Naphthalene, decahydro-4a-methyl-1-methylene-7-(1methylethenyl)-, [4aR-(4a.alpha.,7.alpha.,8a.beta.)] | 17066-67-0 | C_15_H_24_ | 18841971 | 97.5 | 0.86 | 2.87 |
| 81 | 25.4072 | Butanoic acid, 2-methyl-, 2-phenylethyl ester | 24817-51-4 | C_13_H_18_O_2_ | 1795697 | 90.3 | 0.08 | 0.27 |
| 82 | 25.5208 | 1,2-Benzenediol, o-(4-butylbenzoyl)-o'-(2methylbenzoyl) | 1000325-96-0 | C_25_H_24_O_4_ | 1499491 | 73.4 | 0.07 | 0.23 |
| 83 | 25.5629 | Naphthalene,1,2,3,4,4a,5,6,8a-octahydro-4a,8dimethyl-2-(1-methylethenyl)-, [2R(2.alpha.,4a.alpha.,8a.beta.)] | 473-13-2 | C_15_H_24_ | 22194407 | 97.8 | 1.02 | 3.38 |
| 84 | 25.6298 | Epizonarene | 41702-63-0 | C_15_H_24_ | 4150208 | 81.8 | 0.19 | 0.63 |
| 85 | 25.6314 | Zonarene | 41929-05-9 | C_15_H_24_ | 6391765 | 87.8 | 0.29 | 0.97 |
| 86 | 25.6589 | (4-Methylphenyl) methanol, ethyl ether | 1010374-65-1 | C_10_H_14_O | 3208282 | 71.3 | 0.15 | 0.49 |
| 87 | 25.8072 | α-Farnesene | 502-61-4 | C_15_H_24_ | 26614230 | 90.9 | 1.22 | 4.06 |
| 88 | 25.9207 | (R)-1-Methyl-4-(6-methylhept-5-en-2-yl)cyclohexa1,4-diene | 28976-67-2 | C_15_H_24_ | 856506 | 89.2 | 0.04 | 0.13 |
| 89 | 25.9424 | Butylated Hydroxytoluene | 128-37-0 | C_15_H_24_O | 644707 | 84.4 | 0.03 | 0.1 |
| 90 | 26.0293 | Naphthalene,1,2,3,4,4a,5,6,8a-octahydro-7-methyl-4methylene-1-(1-methylethyl)-, (1.alpha.,4a.beta.,8a.alpha.) | 39029-41-9 | C_15_H_24_ | 91850919 | 99.2 | 4.2 | 14 |
| 91 | 26.1125 | (-)-α-Panasinsen | 56633-28-4 | C_15_H_24_ | 784054 | 84.6 | 0.04 | 0.12 |
| 92 | 26.1125 | (2S,4aR,8aR)-4a,8-Dimethyl-2-(prop-1-en-2-yl)1,2,3,4,4a,5,6,8a-octahydronaphthalene | 123123-37-5 | C_15_H_24_ | 828979 | 84.5 | 0.04 | 0.13 |
| 93 | 26.2206 | Naphthalene, 1,2,3,4-tetrahydro-1,6-dimethyl-4-(1methylethyl)-, (1S-cis) | 483-77-2 | C_15_H_22_ | 8902102 | 70.3 | 0.41 | 1.36 |
| 94 | 26.2233 | Naphthalene, 1,2,3,5,6,8a-hexahydro-4,7-dimethyl-1(1-methylethyl)-, (1S-cis) | 483-76-1 | C_15_H_24_ | 24315853 | 97.4 | 1.11 | 3.71 |
| 95 | 26.4036 | (E)-1-Methyl-4-(6-methylhept-5-en-2ylidene)cyclohex-1-ene | 53585-13-0 | C_15_H_24_ | 4103316 | 94.8 | 0.19 | 0.63 |
| 96 | 26.5256 | Naphthalene, 1,2,3,5,6,7,8,8a-octahydro-1,8adimethyl-7-(1-methylethenyl)-, [1R(1.alpha.,7.beta.,8a.alpha.)] | 997297 | C_15_H_24_ | 9235279 | 96 | 0.42 | 1.41 |
| 97 | 26.6811 | Selina-3,7(11)-diene | 6813-21-4 | C_15_H_24_ | 9259320 | 97.1 | 0.42 | 1.41 |
| 98 | 26.7062 | α-Calacorene | 21391-99-1 | C_15_H_20_ | 8561691 | 96.2 | 0.39 | 1.3 |
| 99 | 27.0517 | 1,5-Cyclodecadiene, 1,5-dimethyl-8-(1methylethylidene)-, (E,E) | 15423-57-1 | C_15_H_24_ | 4396858 | 99.4 | 0.2 | 0.67 |
| 100 | 27.1838 | 4-Isopropyl-6-methyl-1-methylene-1,2,3,4tetrahydronaphthalene | 50277-34-4 | C_15_H_20_ | 1511323 | 70.3 | 0.07 | 0.23 |
| 101 | 27.6707 | Caryophyllene oxide | 1139-30-6 | C_15_H_24_O | 10263928 | 98.5 | 0.47 | 1.56 |
| 102 | 27.7822 | 1H-Cycloprop[e]azulen-4-ol, decahydro-1,1,4,7tetramethyl-, [1aR(1a.alpha.,4.beta.,4a.beta.,7.alpha.,7a.beta.,7b.alpha.)] | 552-02-3 | C_15_H_26_O | 5157368 | 94 | 0.24 | 0.79 |
| 103 | 28.0358 | Humulene epoxide I | 19888-33-6 | C_15_H_24_O | 1187500 | 81.3 | 0.05 | 0.18 |
| 104 | 28.2788 | (1R,3E,7E,11R)-1,5,5,8-Tetramethyl-12oxabicyclo[9.1.0]dodeca-3,7-diene | 19888-34-7 | C_15_H_24_O | 3659813 | 97.4 | 0.17 | 0.56 |
| 105 | 28.4059 | 4a(2H)-Naphthalenol, 1,3,4,5,6,8a-hexahydro-4,7dimethyl-1-(1-methylethyl)-, (1S,4R,4aS,8aR) | 19912-67-5 | C_15_H_26_O | 8445521 | 97 | 0.39 | 1.29 |
| 106 | 28.5527 | α-Corocalene | 20129-39-9 | C_15_H_20_ | 1009148 | 94.9 | 0.05 | 0.15 |
| 107 | 28.7021 | 4a(2H)-Naphthalenol,1,3,4,5,6,8a-hexahydro-4,7dimethyl-1-(1-methylethyl)-, (1S,4R,4aS,8aR) | 19912-67-5 | C_15_H_26_O | 1638914 | 87.9 | 0.07 | 0.25 |
| 108 | 28.8202 | 4a(2H)-Naphthalenol,1,3,4,5,6,8a-hexahydro-4,7dimethyl-1-(1-methylethyl)-, (1S,4S,4aS,8aR) | 73365-77-2 | C_15_H_26_O | 2571497 | 87.6 | 0.12 | 0.39 |
| 109 | 29.0233 | .tau.-Cadinol | 1474790 | C_15_H_26_O | 4708378 | 85.2 | 0.22 | 0.72 |
| 110 | 29.058 | Benzenemethanol, 4-methyl-.alpha.-(1-methyl-2propenyl)-, (R*,R*) | 83173-76-6 | C_12_H_16_O | 1142443 | 75.5 | 0.05 | 0.17 |
| 111 | 29.3637 | α-Cadinol | 481-34-5 | C_15_H_26_O | 16980143 | 94.7 | 0.78 | 2.59 |
| 112 | 29.612 | Bisabolol,epi-beta | 235421-59-7 | C_15_H_26_O | 3325657 | 90.6 | 0.15 | 0.51 |
| 113 | 29.6123 | 3-Cyclohexen-1-ol, 1-[(1S)-1,5-dimethyl-4-hexenyl]-4methyl-, (1S) | 15352-77-9 | C_15_H_26_O | 3521753 | 91.6 | 0.16 | 0.54 |
| 114 | 29.741 | Naphthalene, 1,6-dimethyl-4-(1-methylethyl) | 483-78-3 | C_15_H_18_ | 1408638 | 96.5 | 0.06 | 0.21 |
| 115 | 29.9519 | α-Bisabolol | 515-69-5 | C_15_H_26_O | 2521346 | 87.8 | 0.12 | 0.38 |
| 116 | 30.1322 | Bergamotol, Z-.alpha.-trans | 88034-74-6 | C_15_H_24_O | 1751421 | 84.9 | 0.08 | 0.27 |
| 117 | 30.1331 | Isospathulenol | 88395-46-4 | C_15_H_24_O | 1930837 | 85.6 | 0.09 | 0.29 |
| 118 | 30.2828 | 1-Naphthalenol, decahydro-1,4a-dimethyl-7-(1methylethylidene)-, [1R-(1.alpha.,4a.beta.,8a.alpha.)]- | 473-04-1 | C_15_H_26_O | 2411526 | 96 | 0.11 | 0.37 |
| 119 | 30.599 | α-Bisabolol | 515-69-5 | C_15_H_26_O | 2521346 | 87.8 | 0.12 | 0.38 |
| 120 | 30.8591 | 1,4-Azulenediol,1,2,3,3a,4,5,6,8a-octahydro-1,4dimethyl-7-(1-methylethyl)-, (1R,4S) | 2117730-73-9 | C_15_H_26_O_2_ | 1221238 | 79.8 | 0.06 | 0.19 |
| 121 | 31.2368 | Eremophila ketone | 158930-41-7 | C_15_H_24_O | 660400 | 80.9 | 0.03 | 0.1 |
| 122 | 32.4446 | Ambrial | 3243-36-5 | C_16_H_26_O | 1239818 | 93.9 | 0.06 | 0.19 |
| 123 | 38.2805 | 1-Naphthalenemethanol, α-methyl-.α.(phenylmethyl) | 65059-25-8 | C_19_H_18_O | 3608771 | 71.7 | 0.17 | 0.55 |
| 124 | 40.7319 | Dihydroyashabushiketol, Ac derivative | 88815-02-5 | C_21_H_24_O_3_ | 1539336 | 81.2 | 0.07 | 0.23 |
| 125 | 41.6605 | 1,7-Diphenyl-4-hepten-3-one | 79559-59-4 | C_19_H_20_O | 10398564 | 98.2 | 0.48 | 1.58 |
